# Improving Parkinson’s disease diagnosis by non-invasive detection of retinal biomarkers in MPTP monkeys using ERG and pupillometry

**DOI:** 10.1038/s41531-026-01391-y

**Published:** 2026-06-03

**Authors:** Jonathan Munro, Andrée-Anne Lavigne, Shirley Fecteau, Thérèse Di Paolo, Marc Hébert, Martin Parent

**Affiliations:** 1 CERVO Brain Research Center, Université Laval, Quebec City, QC Canada; 2 CHU de Québec Research Center, Université Laval, Quebec City, QC Canada

**Keywords:** Diseases, Neurology, Neuroscience

## Abstract

Parkinson’s disease (PD) diagnosis is currently made by clinical observation of motor symptoms when around 50% of dopamine neurons in the substantia nigra pars compacta are already lost. Non-motor symptoms, such as vision problems, occur earlier during disease progression and could be caused by altered retinal functioning. Various techniques exist to detect retinal alterations and, therefore, could provide biomarkers for earlier diagnosis of PD. The aim of this project is to determine potential retinal biomarkers for PD by using electroretinography (ERG) and pupillometry. In vivo measurements were performed on four non-human primates, before and after they were rendered parkinsonian by administration of 1-méthyl-4-phényl-1,2,3,6-tetrahydropyridine (MPTP), a neurotoxin that induces degeneration of dopamine neurons. ERG results showed a significant increase in photopic b-wave implicit time and reduced oscillatory potential (OP) amplitudes in both photopic and scotopic conditions following MPTP administration. The post-illumination pupillary response showed a consistently larger pupil diameter post-MPTP. Post-mortem examination revealed a significant thinning of the outer nuclear retinal layer. Tyrosine hydroxylase and melanopsin-containing cells were reduced in MPTP-intoxicated monkeys. Altogether, these results indicate that MPTP intoxication leads to functional retinal changes detectable by ERG and pupillometry, possibly attributed to cellular alterations.

## Introduction

Parkinson’s disease (PD) is a neurodegenerative condition characterized by the progressive degeneration of dopaminergic (DA) neurons in the substantia nigra pars compacta (SNc). The SNc is an important midbrain nucleus participating in the function of the basal ganglia, a set of subcortical structures involved in motor behaviour. The loss of DA modulation following degeneration of SNc neurons results in the expression of the cardinal symptoms of PD, namely bradykinesia, rigidity, resting tremor and postural instability^1^. It is upon the clinical presentation of these motor symptoms that PD is typically diagnosed. Currently, no other diagnostic method is viable^1^. Once these motor symptoms become apparent, around 50% of the DA neurons of the SNc have degenerated^2^. Until now, this damage is considered irreversible, and therefore presents a problem: once diagnosed, it is too late to implement interventions that could potentially reduce or stop the rate of degeneration. Many such potential interventions are being investigated^3,4^, however, their use depends upon diagnosis of the disease much earlier in its progression. As the second most common neurodegenerative condition worldwide, PD is projected to increase in prevalence and burden healthcare systems^5^. This underscores the importance of developing methods for earlier diagnosis to allow treatment before the disease becomes too severe. Diagnosing PD only according to motor symptoms also presents the potential for misdiagnosis. Other diseases share similar motor symptoms with PD such as essential tremor and Lewy body dementia. This could contribute to an incorrect diagnosis in about 25% of patients, particularly those with early onset PD^6–8^. Therefore, while diagnosing PD early is important, accurate diagnosis is still paramount for the implementation of appropriate treatments. For this reason, it is essential that other biomarkers are discovered that will allow for greater sensitivity and specificity in its diagnosis.

In addition to the motor symptoms, many non-motor symptoms are often experienced by patients with PD^9^. These symptoms become noticeable prior to the onset of the motor symptoms and will increase in severity with the progression of the disease. Vision problems such as changes in visual acuity and contrast sensitivity are often reported, supporting the idea that PD may be causing changes to the retina as well as the brain^10–15^. Furthermore, these changes seem to occur early enough that they could become biomarkers for diagnosis prior to the onset of motor symptoms^1,16^. Therefore, if retinal biomarkers are determined and can be detected during the prodromal stages of the disease, this could provide the opportunity for treatments that could delay disease progression^3,17,18^.

As a part of the central nervous system, there is increasing interest in the retina as a source of biomarkers for neurological conditions. Non-invasive approaches already exist to assess the anatomy and physiology of the retina. Using such methods, noticeable differences were observed in the activity and morphology of the retina between healthy individuals and those with neurological or psychiatric conditions such as attention-deficit/hyperactivity disorder, autism spectrum disorder, Alzheimer’s disease, schizophrenia, bipolar disorder, major depression, multiple sclerosis, and PD^10,19–26^. This provides some indication that while these conditions alter the brain, changes may also extend to the retina and would therefore be detectable at the eye level using non-invasive methods. If specificity in these retinal changes could be found for each condition, the retina could then become an important source of biomarkers for reliable diagnosis.

Electroretinography (ERG) is a tool that allows the recording of the summed electrical response of retinal neurons evoked by brief flashes of light^27^. The ERG waveform can be divided into 2 main waves. The first is the a-wave, a negative deflection that reflects the activity of the photoreceptors^28,29^. The second is the b-wave, a large positive component that follows the a-wave and reflects the activity of the bipolar cells with some influence from the Müller glial cells^30,31^. Using a red flash over a blue background light, a third wave called the photopic negative response (PhNR) can be measured following the b-wave and reflects the activity of the ganglion cells (GC)^32^. Both the amplitudes of positive peaks and negative troughs in the ERG waveform, along with their corresponding latencies (implicit times), can be quantified^21,27^. In addition to these components, oscillatory potentials (OPs) can be analyzed. OPs consist of small, high-frequency wavelets superimposed on the ascending limb of the b-wave and are thought to reflect inner-retinal activity, including contributions from DA amacrine cells (ACs)^33–35^. Distinct ERG protocols, based on light adaptation state and stimulus characteristics, enable the selective evaluation of rod-mediated (scotopic ERG), cone-mediated (photopic ERG), and ganglion cell-driven retinal responses (PhNR). Furthermore, recordings can be made over a range of flash intensities to produce a luminance response function, from which variables can be extracted, such as the Vmax, corresponding to the maximum b-wave amplitude^36–38^. Using this technique, differences in ERG parameters have been observed in PD patients when compared to control individuals^39–46^. However, results have been inconsistent, likely due to substantial interindividual variability. Findings have included no change to the a- or b-wave amplitude or implicit time^47,48^, changes to both waves^39–41,44,45^, or to the b-wave alone^42,43,46^.

Another technique providing an indirect measure of retinal function is pupillometry, which quantifies dynamic changes in pupil diameter in response to light stimulation. The pupillary light reflex reflects the integrated activity of rods and cones together with intrinsically photosensitive retinal ganglion cells (ipRGCs). Notably, short-wavelength (blue) light strongly activates melanopsin-expressing ipRGCs and preferentially drives the sustained component of the pupillary response, including the post-illumination pupil response^49,50^. It is known that melanopsin expression is influenced by DA in the retina^51^. Reduced retinal DA has been observed in the retina of human PD patients and of animals modelling PD^52–55^. This suggests that the pupillary light reflex may be altered in PD by impaired melanopsin expression.

Current research looking into retinal changes in PD are primarily being conducted in humans and rodents. Although changes have been found in rodent models of PD, such research cannot be deemed reflective of what would be seen in humans, in part because of significant interspecies differences between the rodent and primate retina^56^. On the other hand, human research is often limited by poor control over interindividual variability, such as confounding health conditions, medications and lifestyle factors, and by challenges related to comparisons between in vivo results and post-mortem retina and brain analyses. Moreover, most studies use only one recording technique, limiting cross-technique comparisons and the identification of potential correlations. Because specificity remains a major challenge for retinal biomarkers in PD, integrating multiple techniques to obtain a broader biomarker profile is essential.

This study aims to identify retinal biomarkers of PD using ERG and pupillometry in a non-human primate (NHP) model, the closest analogue to human PD. By applying both techniques to the same animals and comparing pre- and post-PD conditions, we controlled for interindividual variability and explored potential links between ERG and pupillary changes. Finally, post-mortem analyses were performed on the retina and brains of the animals to find potential correlations between in vivo functional changes of the retina and post-mortem morphological and neurochemical alterations.

## Results

### Evaluation of DA lesion by motor scoring and DA metabolite concentration

The MPTP monkeys scored between 6.72 and 9.41 out of 16.00 (mean of 8.34 ± 1.30) on a well-established motor disability scale^60^ indicating moderate to severe parkinsonian symptoms (Table 1). Their motor score remained stable for the remainder of the experiment. Analysis of the CSF was made using high-performance liquid chromatography to determine changes in levels of the DA metabolite HVA. Five months following MPTP administration, the mean HVA level was reduced by 66.83% (162.11 ± 22.01 vs. 488.68 ± 162.00 ng/ml). PostMPTP administration of L-dopa induced an 89.27% increase in HVA (306.83 ± 49.62 vs. 162.11 ± 22.01 ng/ml), but this was still 37.21% lower than preMPTP levels (306.83 ± 49.62 vs. 488.68 ± 162.00). Average HVA levels remained similar across recording sessions of a given experimental condition.

**Table 1:** Information on MPTP-intoxicated and control NHPs

Animal ID	Group	Age (years)	Sex	Weight (kg)	Motor disability score (/16)*	Total MPTP (mg)
**CTL1**	Control	3	Male	2.9	NA	NA
**CTL2**	Control	2	Male	2.9	NA	NA
**CTL3**	Control	2	Male	2.5	NA	NA
**CTL4**	Control	2	Male	2.5	NA	NA
**CTL5**	Control	4	Female	2.9	NA	NA
**CTL6**	Control	4	Female	2.7	NA	NA
**CTL7**	Control	4	Female	2.6	NA	NA
**CTL8**	Control	6	Female	3.4	NA	NA
**MPTP1**	MPTP	5	Female	3.2	9.41	14.11
**MPTP2**	MPTP	5	Female	3.3	9.37	9.46
**MPTP3**	MPTP	5	Female	3.2	6.72	9.91
**MPTP4**	MPTP	5	Female	3.6	7.85	12.67

^*^Motor disability was assessed using the scale outlined in Hadj Tahar et al. (2004).

### Evaluation of DA lesion in the SNc and striatum

**Fig. 1:**
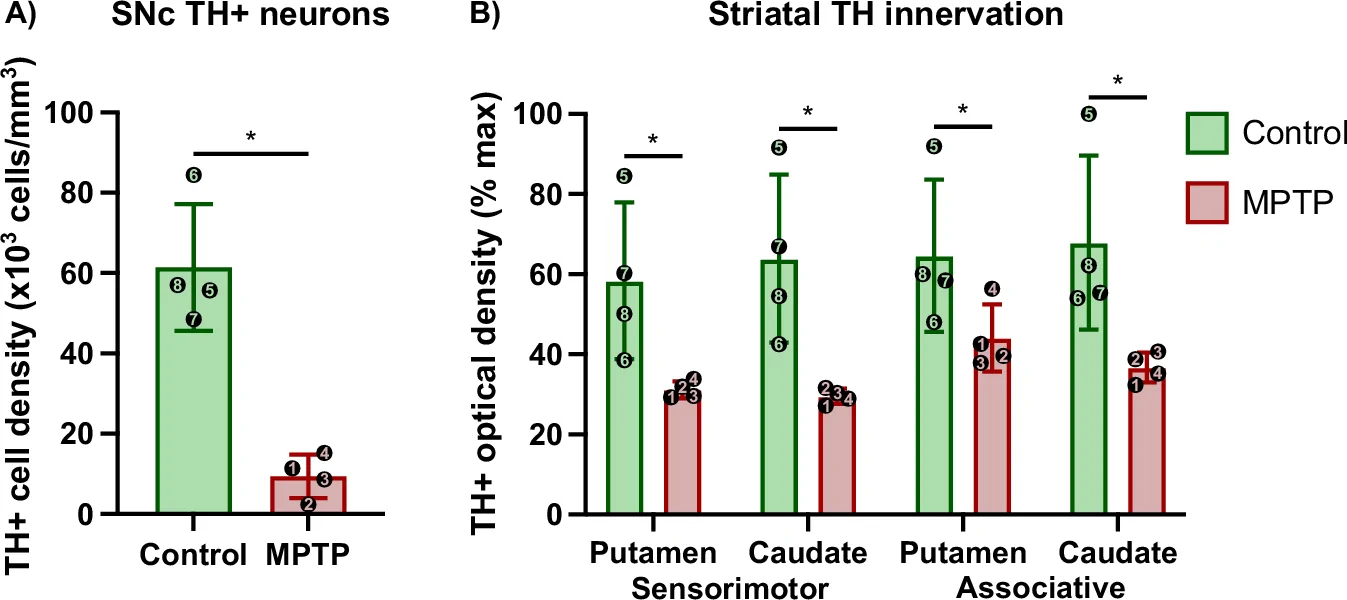
The effect of MPTP on dopaminergic neurons in the SNc and their striatal innervation. **A** The average densities of TH+ cells in the SNc between control and MPTP-intoxicated monkeys. **B** The average fluorescent optical density of TH in the sensorimotor and associative functional regions of the putamen and caudate nucleus. Bars represent the mean with SD. *p-value < 0.05. The numbered dots represent individual values with NHP numbers as presented in Table 1.

Average MPTP-induced DA cell loss in the SNc was determined by comparing estimated densities of TH+ neurons in the SNc of MPTP (MPTP1-4) and control (CTL5-8) monkeys, as obtained by unbiased stereology. In control monkeys, the estimated population of TH+ neurons was 61.43 ± 15.81 × 10^3^ compared to 9.40 ± 5.40 × 10^3^ in MPTP-intoxicated monkeys, showing an 84.71% lower estimated population (*P* = 0.01) (Fig. 1A). To quantify striatal DA denervation, comparisons of the average TH optical densities were made between MPTP and the same cohort of control monkeys. MPTP-intoxicated monkeys showed a 50.35% (*P* = 0.01) and 38.97% (*P* = 0.01) lower TH immunoreactivity in the sensorimotor and associative striatal territories respectively (Fig. 1B). More precisely, in the caudate nucleus we observed a 53.79% (*P* = 0.01) and 45.84% (*P* = 0.01) lower immunoreactivity in the sensorimotor and associative territories, respectively, compared to 46.59% (*P* = 0.01) and 31.74% (*P* = 0.02) in the putamen. The lower TH immunoreactivity observed in MPTP monkeys appeared homogenous throughout the anteroposterior axis of the striatum, with the exception of the associative territory of the putamen, for which the pre-commissural and commissural levels showed less pronounced DA denervation, with only 19.93% and 28.08% lower immunoreactivity, respectively, compared to 47.28% observed at the post-commissural level. DA denervation was not observed in the striatal limbic territory (nucleus accumbens) of the MPTP-intoxicated monkeys.

### MPTP impact on photopic ERG

**Fig. 2:**
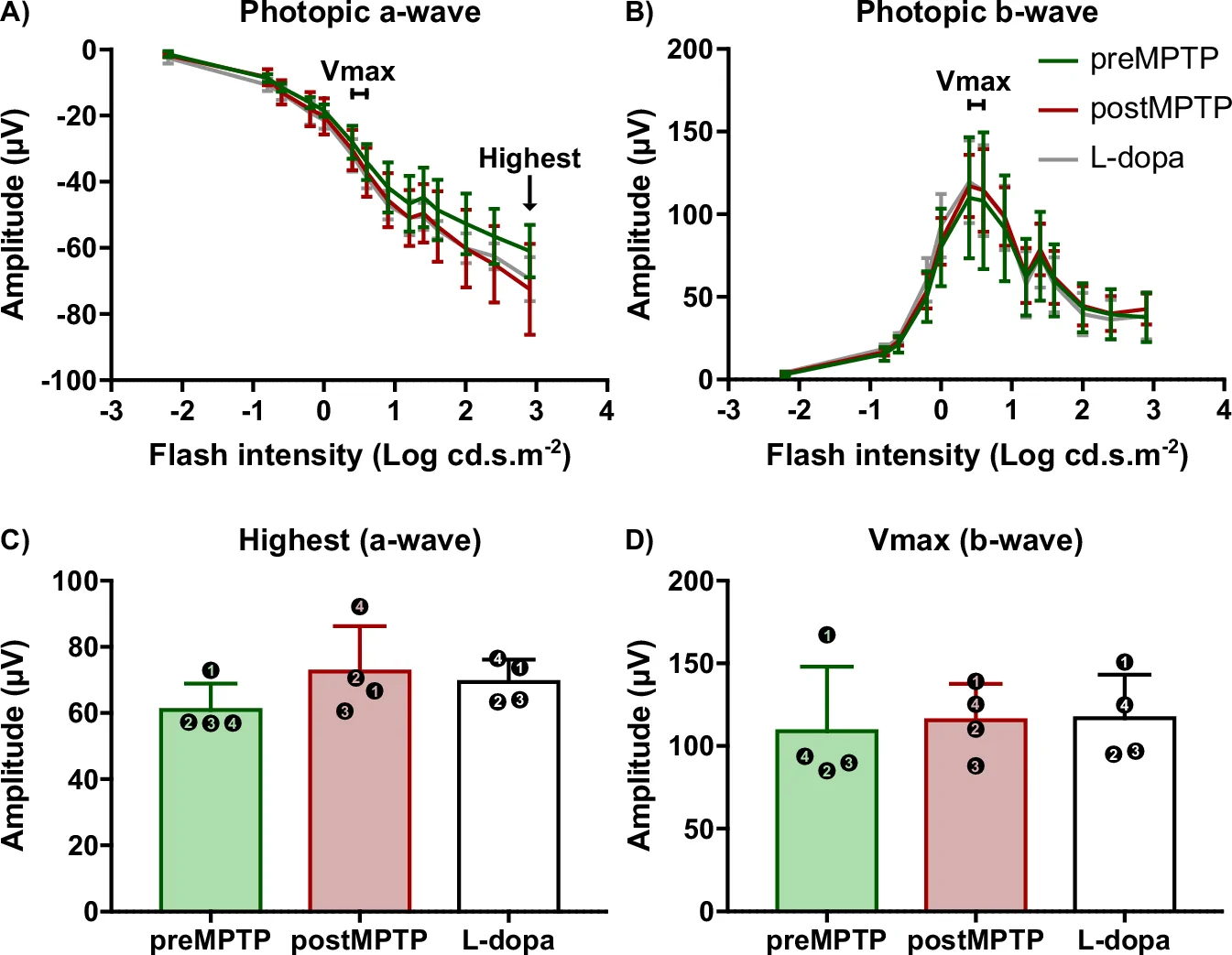
Average photopic ERG amplitudes of a-waves and b-waves between each experimental condition. **A, B** Luminance response functions for each wave. **C, D** Highest and Vmax amplitudes for the a-wave and b-wave, respectively. Amplitudes are displayed as the mean with SD of the four animals for each light flash intensity (**A**, **B**) and for the highest a-wave (**C**) or b-wave Vmax (**D**) flash intensities (**B**). The numbered dots represent individual values with NHP numbers as presented in Table 1.

Following assessment of the DA lesion, we investigated if MPTP altered the ERG response. Analysis of the photopic a- and b-wave amplitudes showed no significant differences between any of the experimental conditions (Figs. 2A, B, D. Tables 2, 3). The photopic b-wave implicit time at Vmax was significantly prolonged by 3.57% in the postMPTP condition compared with preMPTP (*P* = 0.02, *g* = 1.35). The a-wave implicit time at Vmax showed a trend toward prolongation in the postMPTP condition relative to preMPTP (3.77% increase; *P* = 0.10, *g* = 1.61). In contrast, L-dopa treatment was associated with a trend toward shortened a-wave implicit time at Vmax compared with postMPTP (*P* = 0.10, *g* = −0.76), restoring the a-wave timing to that observed in the preMPTP condition. A larger increase in a-wave amplitude (18.93% postMPTP vs. preMPTP) was observed at the highest flash intensity (2.9 log cd·s·m⁻²), although the difference did not reach significance and the effect size was small (Fig. 2C. Table 3).

**Table 2:** Calculated ERG values for each condition, with comparisons between condition for each ERG parameter

	Mean (SD)			% Difference		
ERG parameters	preMPTP	postMPTP	L-dopa	postMPTP vs. preMPTP	L-dopa vs. postMPTP	L-dopa vs. preMPTP
*Photopic*						
a-Wave amplitude (μV)						
Vmax	−31.06 (5.19)	−33.74 (6.73)	−35.34 (4.09)	8.63	4.72	13.76
Highest (2.9 Log)	−61.00 (7.94)	−72.55 (13.74)	−69.42 (6.70)	18.93	−4.30	13.81
b-Wave amplitude (μV)						
Vmax	108.98 (38.99)	115.73 (21.88)	116.96 (26.33)	6.20	1.06	7.32
a-Wave implicit time (ms)						
Vmax	13.27 (0.76)	13.77 (0.75)	13.31 (0.52)	3.77	−3.33	0.31
Highest (2.9 Log)	10.79 (0.79)	11.21 (0.85)	10.75 (0.50)	3.86	−4.09	−0.39
b-Wave implicit time (ms)						
Vmax	28.04 (0.72)	29.04 (0.95)	28.63 (0.32)	**3.57**	−1.43	2.08
PhNR amplitude (μV)						
From baseline	82.26 (8.16)	92.52 (21.95)	95.58 (21.75)	12.48	3.31	16.20
From b-Wave peak	138.49 (16.58)	152.61 (30.36)	156.47 (32.63)	10.20	2.53	12.98
PhNR implicit time (ms)						
From baseline	57.75 (1.50)	58.67 (1.40)	56.88 (1.44)	1.59	−3.05	−1.52
OP amplitude (μV)						
OP1	6.33 (2.26)	5.67 (0.86)	7.05 (1.11)	−10.38	24.18	11.30
OP2	7.60 (3.03)	5.45 (1.67)	9.52 (3.65)	−28.25	**74.57**	25.25
OP3	15.28 (6.46)	11.44 (2.87)	17.68 (3.31)	−25.11	54.51	15.71
Sum	29.21 (11.48)	22.57 (5.10)	34.25 (7.88)	−22.73	51.73	17.24
OP implicit time (ms)						
OP1	15.75 (0.50)	16.00 (0.82)	15.75 (0.50)	1.59	−1.56	0.00
OP2	20.75 (0.96)	20.75 (1.50)	20.75 (0.96)	0.00	0.00	0.00
OP3	26.00 (0.82)	26.50 (1.00)	26.25 (0.50)	1.92	−0.94	0.96
*Scotopic*						
a-Wave amplitude (μV)						
Mixed rods/cones (Vmax)	−157.10 (33.29)	−172.17 (25.76)	−177.28 (23.68)	9.60	2.97	12.85
Pure rods (-1 Log)	−35.62 (9.03)	−39.05 (15.74)	−37.26 (5.25)	9.62	−4.58	4.60
Highest (2 Log)	−191.78 (39.23)	−212.33 (33.58)	−214.61 (39.67)	10.71	1.08	11.91
b-Wave amplitude (μV)						
Mixed rods/cones (Vmax)	223.42 (49.26)	236.22 (41.00)	244.98 (40.95)	5.73	3.71	9.65
Pure rods (-1 Log)	169.43 (35.67)	178.82 (33.41)	185.10 (31.29)	5.54	3.51	9.25
a-Wave implicit time (ms)						
Mixed rods/cones (Vmax)	14.32 (0.80)	14.99 (0.60)	14.19 (0.38)	4.65	−5.35	−0.95
Pure rods (-1 Log)	21.71 (0.91)	23.00 (0.97)	22.13 (0.95)	5.95	−3.80	1.92
Highest (2 Log)	8.96 (0.70)	9.67 (0.00)	9.00 (0.41)	7.91	−6.90	0.47
b-Wave implicit time (ms)						
Mixed rods/cones (Vmax)	36.77 (1.06)	36.48 (1.09)	34.31 (1.45)	−0.79	−5.94	-6.69
Pure rods (-1 Log)	52.75 (5.06)	54.63 (2.72)	48.50 (5.28)	3.55	−**11.21**	-8.06
OP amplitude (μV)						
OP1	21.93 (7.05)	20.66 (4.06)	23.93 (2.17)	-5.80	15.81	9.09
OP2	13.74 (6.48)	11.95 (2.54)	17.35 (2.35)	-13.06	45.21	26.25
OP3	3.56 (5.82)	2.02 (2.20)	5.58 (3.52)	-43.22	176.06	56.74
Sum	39.23 (16.73)	34.63 (8.28)	46.86 (7.18)	-11.74	35.31	19.43
OP implicit time (ms)						
OP1	12.00 (0.00)	12.25 (0.50)	12.50 (0.58)	2.08	2.04	4.17
OP2	20.00 (0.82)	20.25 (1.26)	19.75 (0.50)	1.25	-2.47	−1.25
OP3	26.50 (1.29)	26.25 (1.71)	25.50 (1.29)	−0.94	-2.86	−3.77

Bolded values indicate statistical significance (*P* < 0.05).

**Table 3:** Effect size comparisons between condition for each ERG parameter

	Effect size (p-value)		
ERG parameters	postMPTP vs. preMPTP	L-dopa vs. postMPTP	L-dopa vs. preMPTP
*Photopic*			
a-Wave amplitude (μV)			
Vmax	0.24 (>0.99)	0.34 (>0.99)	0.53 (>0.99)
Highest (2.9 Log)	0.47 (0.47)	−0.18 (> 0.99)	**1.06** (0.23)
b-Wave amplitude (μV)			
Vmax	0.18 (>0.99)	0.07 (>0.99)	0.30 (>0.99)
a-Wave implicit time (ms)			
Vmax	**1.61** (0.10)	−0.76 (0.10)	0.09 (>0.99)
Highest (2.9 Log)	0.61 (0.87)	−0.53 (0.87)	−0.09 (>0.99)
b-Wave implicit time (ms)			
Vmax	**1.35** (**0.02**)	−0.45 (0.87)	**1.04** (0.33)
PhNR amplitude (μV)			
From baseline	0.44 (>0.99)	0.31 (>0.99)	0.64 (0.47)
From b-Wave peak	0.40 (>0.99)	0.24 (>0.99)	0.56 (0.47)
PhNR implicit time (ms)			
From baseline	0.90 (>0.99)	−**2.97** (0.07)	−0.75 (0.47)
OP amplitude (μV)			
OP1	−0.24 (>0.99)	**2.21** (0.23)	0.32 (0.47)
OP2	−**1.12** (0.47)	**1.27** (**0.01**)	0.69 (0.47)
OP3	−0.73 (0.87)	**1.62** (0.10)	0.33 (0.87)
Sum	−0.70 (0.87)	**1.77** (0.10)	0.44 (0.87)
OP implicit time (ms)			
OP1	0.19 (>0.99)	−0.19 (>0.99)	0.00 (>0.99)
OP2	0.00 (>0.99)	0.00 (>0.99)	0.00 (>0.99)
OP3	0.63 (0.87)	−0.36 (>0.99)	0.36 (>0.99)
*Scotopic*			
a-Wave amplitude (μV)			
Mixed rods/cones (Vmax)	0.45 (0.47)	0.27 (>0.99)	0.91 (0.23)
Pure rods (-1 Log)	0.22 ( > 0.99)	−0.11 (0.87)	0.28 (0.87)
Highest (2 Log)	0.75 (0.10)	0.12 (>0.99)	**1.39** (0.10)
b-Wave amplitude (μV)			
Mixed rods/cones (Vmax)	0.50 (0.87)	0.35 (0.87)	0.56 (0.10)
Pure rods (-1 Log)	0.42 (>0.99)	0.33 (0.87)	0.44 (0.87)
a-Wave implicit time (ms)			
Mixed rods/cones (Vmax)	**0.80** (>0.99)	−0.65 (0.47)	−0.11 (>0.99)
Pure rods (-1 Log)	**0.88** (0.07)	−**1.85** (0.16)	0.23 (>0.99)
Highest (2 Log)	0.74 (0.47)	−**1.19** (>0.99)	0.04 (0.23)
b-Wave implicit time (ms)			
Mixed rods/cones (Vmax)	−0.13 ( >0.99)	**−0.92** (0.23)	**−0.83** (0.47)
Pure rods (-1 Log)	0.49 (>0.99)	−**1.70** (**0.04**)	−**1.54** (0.23)
OP amplitude (μV)			
OP1	−0.24 (>0.99)	**0.85** (0.47)	**0.23** (>0.99)
OP2	−0.26 (0.87)	**1.67** (0.10)	**0.55** (0.87)
OP3	−0.25 ( > 0.99)	**0.98** (0.23)	**0.61** (0.47)
Sum	−0.32 (>0.99)	**1.55** (0.47)	**0.51** (>0.99)
OP implicit time (ms)			
OP1	0.36 (>0.99)	0.19 (>0.99)	0.63 (0.87)
OP2	0.36 (>0.99)	−0.36 (>0.99)	−0.36 (>0.99)
OP3	−0.36 (>0.99)	−0.57 (0.87)	**−0.89** (0.33)

Bolded values indicate large effect sizes or statistically significant *p*-values (*P* < 0.05).

### MPTP impact on scotopic ERG

**Fig. 3:**
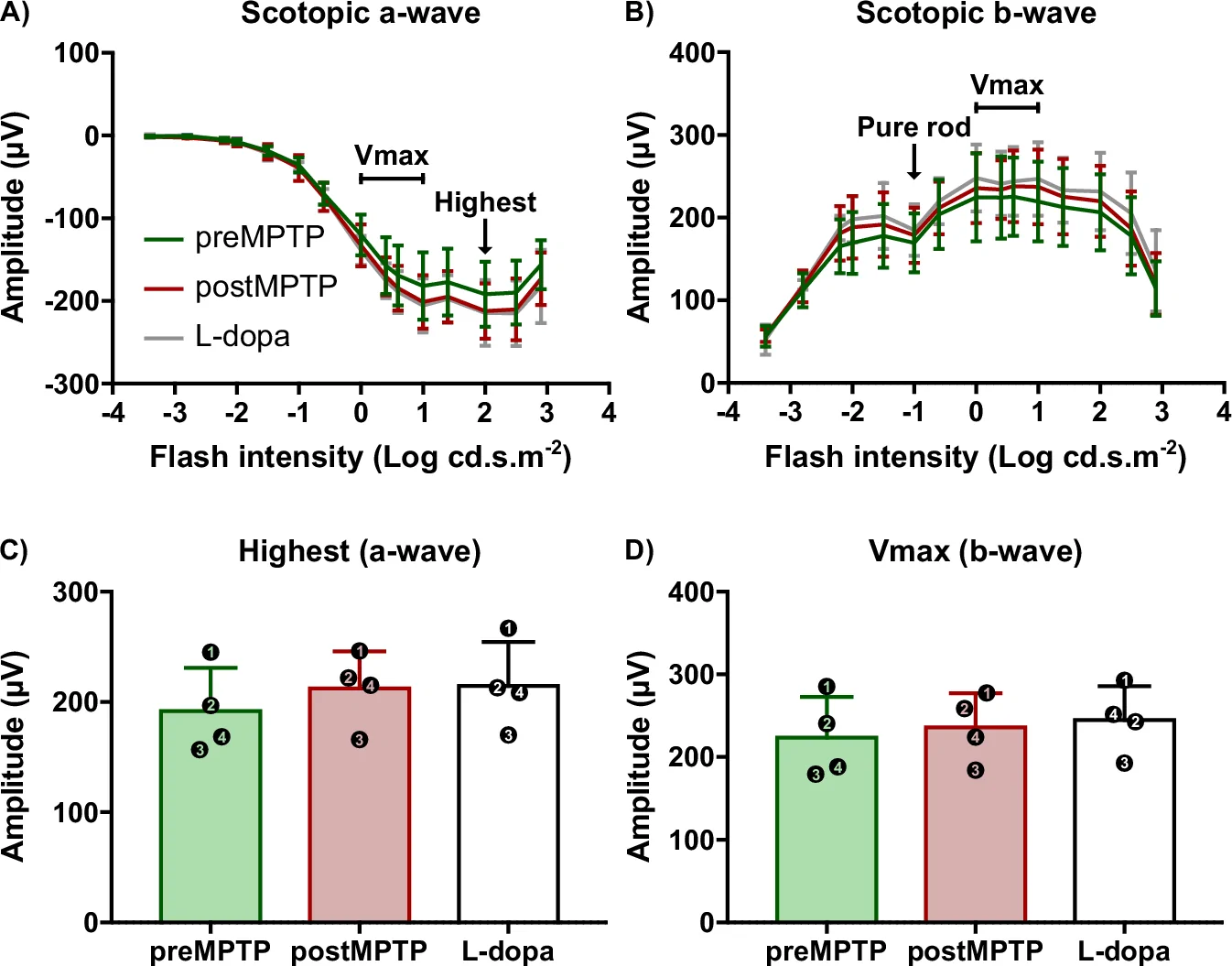
Average scotopic ERG amplitudes of a-waves and b-waves between each experimental condition. **A, B,** Luminance response functions for each wave. **C, D** Highest and Vmax amplitudes for the a-wave and b-wave, respectively. Amplitudes are displayed as the mean with SD of the four animals for each light flash intensity (**A**, **B**) and for the highest a-wave (**C**) or b-wave Vmax (**D**) flash intensities. The numbered dots represent individual values with NHP numbers as presented in Table 1.

Analysis of the scotopic condition involved looking at MPTP-induced alterations to the Vmax, considered to be a mixed rod-cone response, and at the -1 Log cd·s·m^−2^ flash intensity, considered to be the pure rod response. Analysis was also performed at the highest a-wave amplitude that occurred at 2 log cd·s·m⁻². At Vmax, analysis of the scotopic a- and b-wave amplitudes and implicit times showed no significant differences between any of the conditions (Fig. 3A, B, D. Table 2). Although trends were observed for a prolonged pure rod a-wave implicit time (*P* = 0.07, *g* = 0.88) and an increased amplitude for the highest a-wave intensity (2 log cd·s·m⁻²) (*P* = 0.10, *g* = 0.75). Relative to the post-MPTP condition, L-dopa administration significantly reduced the pure rod b-wave implicit time by 11.21% (48.50 ± 5.28 ms vs. 54.63 ± 2.72 ms, *P* = 0.04, *g* = −1.70) (Table 3).

### MPTP impact on the ERG photopic negative response

**Fig. 4:**
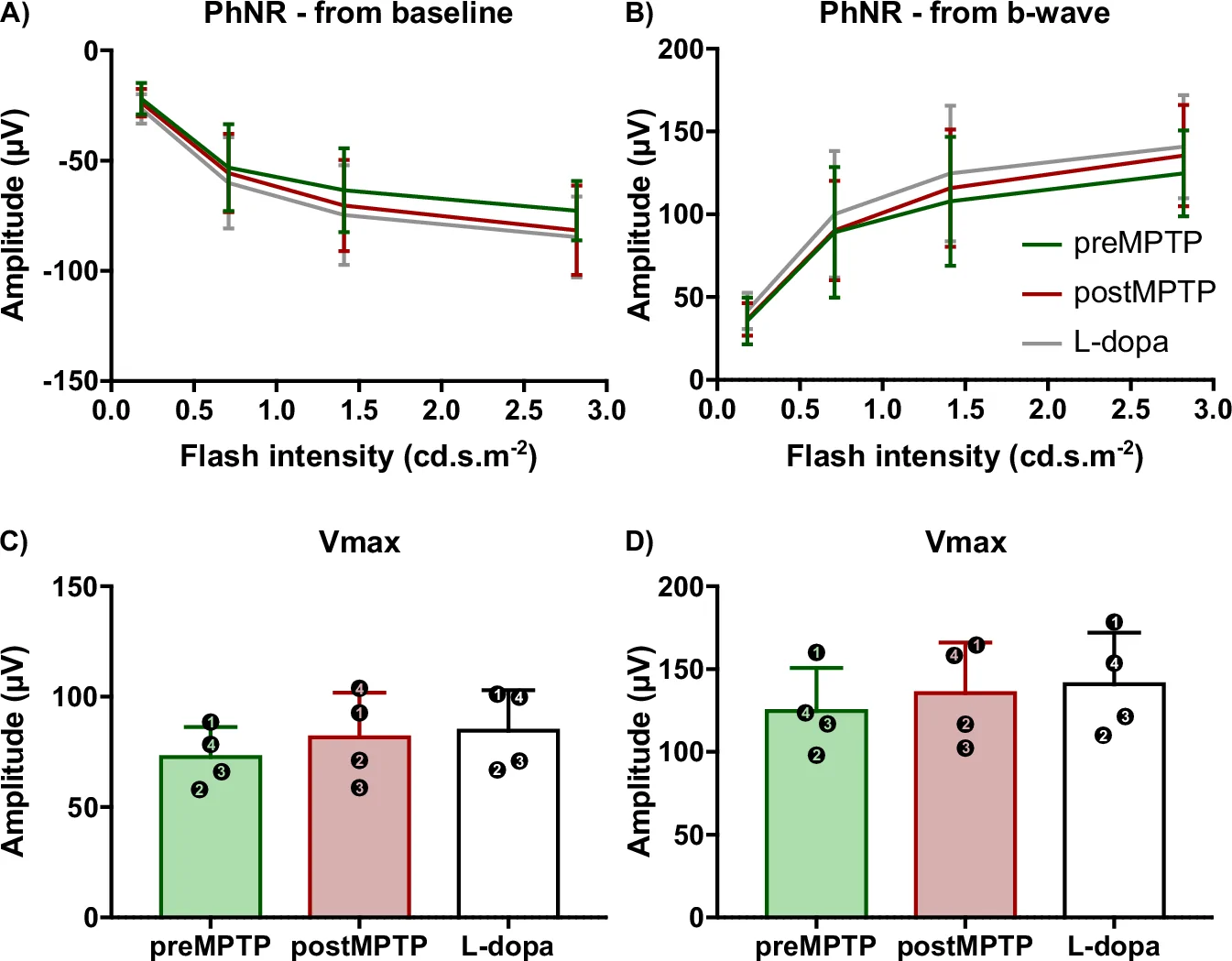
Average PhNR ERG amplitudes between each experimental condition. **A, B** Luminance response functions for each PhNR amplitude calculation. **C, D** Vmax amplitudes for each PhNR amplitude calculation. Amplitudes were calculated from 0 (**A**, **C**) and from the peak of the b-wave amplitude (**B**, **D**). Amplitudes are displayed as the mean with SD of the four animals for each light flash intensity (**A, B**) and for the Vmax (**C**, **D**). The numbered dots represent the individual values with NHP numbers as presented in Table 1.

Amplitudes of the PhNR wave can be calculated relative to the baseline or the peak of the b-wave. Given that saturation of the GCs was not observed across the 4 light intensities, PhNR amplitudes from the highest flash intensity were selected for analysis. From both methods of calculation, no statistically significant difference was observed between the experimental conditions for PhNR amplitudes. However, a trend for a reduced implicit time was observed after L-dopa treatment when compared to postMPTP (*P* = 0.07, *g* = −2.97) (Fig. 4A–D, Table 2).

### MPTP impact on the ERG oscillatory potentials

**Fig. 5:**
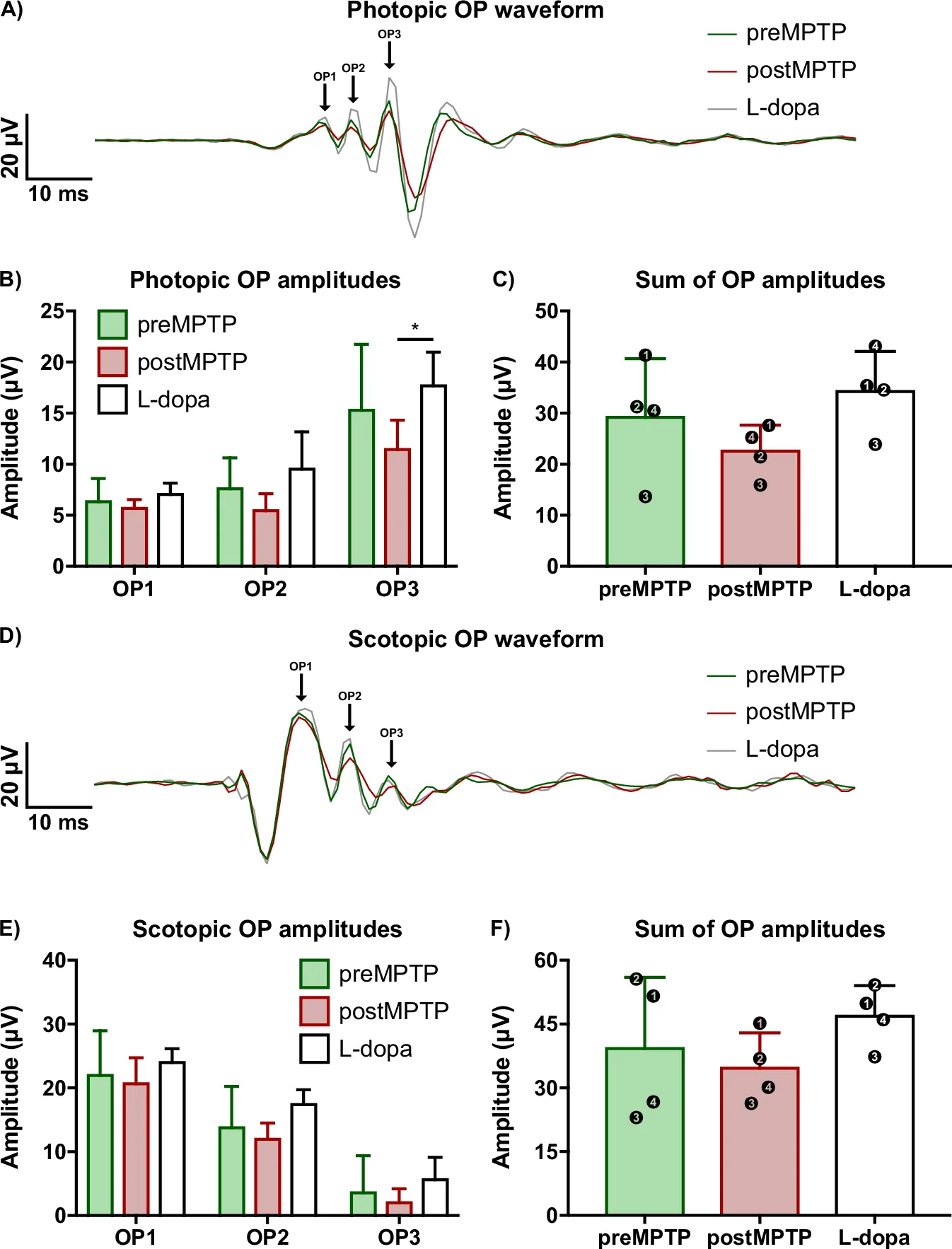
Comparison of photopic and scotopic OP measurements. **A**, **D** Average OP waveforms between each experimental condition. Arrows point to the OPs from which the peak amplitudes were used for further analysis. **B**, **E** Average peak amplitudes between conditions of each OP. **C**, **F** Average sum of OP amplitudes from each animal between conditions. Each numbered dot represents the individual value with the NHP number as represented in Table 1. All bars illustrate the mean and SD data from the four animals.

To investigate MPTP-induced changes to OP amplitudes, comparisons were made for the averages of OP1, OP2 and OP3 separately and for the average sum of OPs between experimental conditions. Under the photopic condition, OP1, OP2 and OP3 amplitudes show reduction following MPTP administration, without statistical significance but with Hedge’s *g* scores indicating a medium to large effect sizes for OP2 and OP3 (Fig. 5A, B. Tables 2, 3). L-dopa administration increased individual OP amplitudes from post-MPTP levels, with large to very large effect sizes, reaching significance for OP2 (74.57% increase, *P* = 0.01, *g* = 1.27), a trend towards significance for OP3 (54.51% increase, *P* = 0.10, *g* = 1.62), and with amplitudes also surpassing the preMPTP levels. The average sum of OPs showed a non-significant reduction after MPTP intoxication (medium effect size) followed by a trend for an increase of 51.73% (*P* = 0.10, *g* = 1.77) upon L-dopa administration (Fig. 5C). No changes were observed to the OP implicit times (Table 2).

Similar results were obtained under the scotopic condition, with MPTP intoxication leading to non-significant (small effect sizes) reductions in individual OP amplitudes (Figs. 5D, E. Tables 2, 3). L-dopa administration increased individual OP amplitudes from post-MPTP levels, with large to very large effect sizes, reaching a trend towards significance for OP2 only (45.21% increase, *P* = 0.10, *g* = 1.67) and with amplitudes also surpassing the pre-MPTP levels. Similarly, the average sum of OPs was reduced, but not significantly (small effect size), after MPTP intoxication followed by an increase upon L-dopa administration with a very large effect size (Fig. 5F). No changes were observed to the OP implicit times (Table 2).

### MPTP impact on the pupillary reflex

**Fig. 6:**
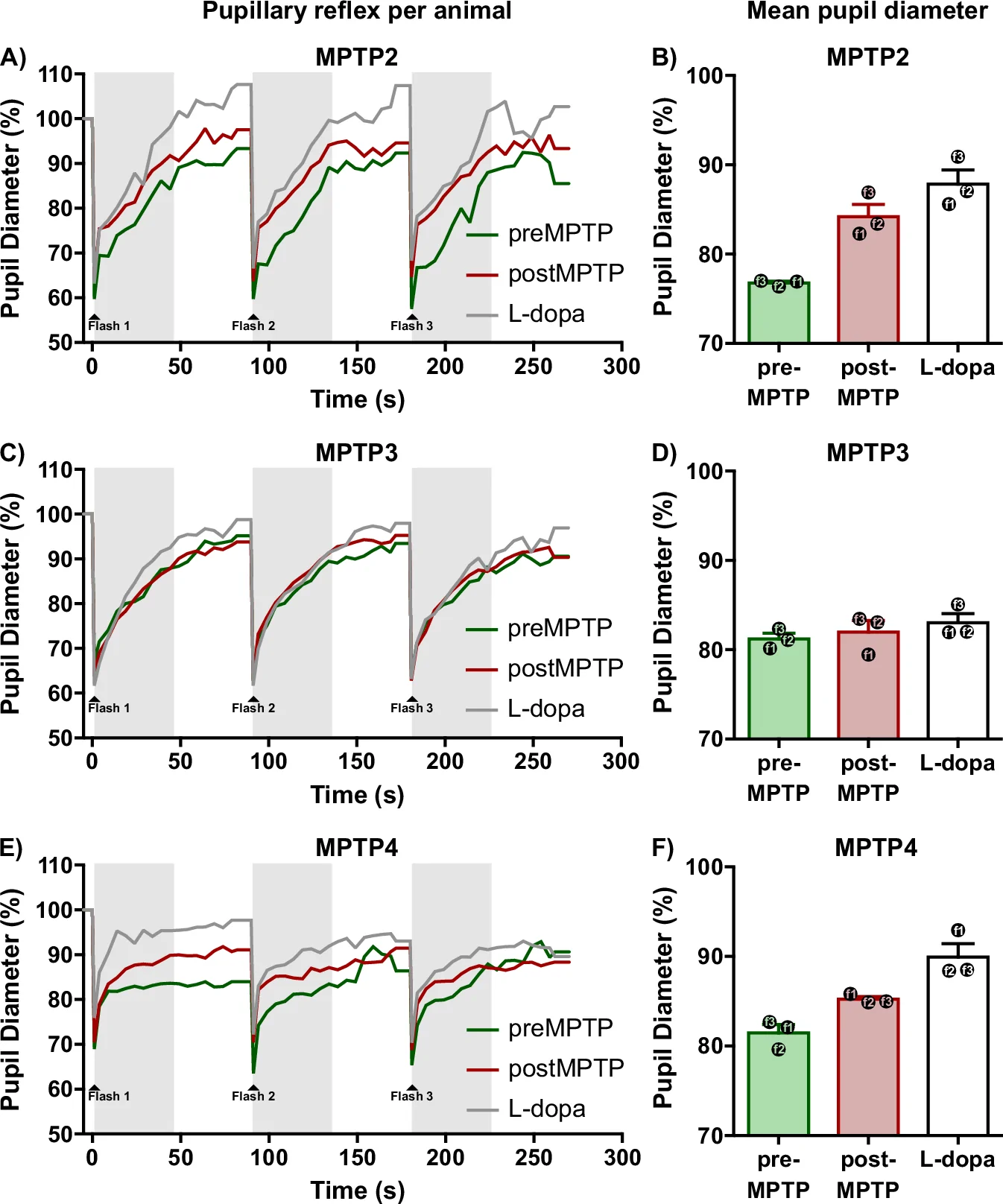
Pupillary reflex between experimental conditions. **A**, **C**, **E** Individual pupillary response curves from 3 NHPs (MPTP2-4) for each experimental condition. Pupil diameter measurements were made continuously for 270 seconds, during which blue light flashes occurred at 0, 90 and 180 seconds, illustrated by the arrows. Pupil diameters are presented as the percent of the average pupil diameter during the 5 seconds prior to the first flash. The grey rectangular portions of the background show the ranges of pupil diameters that were averaged for quantification. **B**, **D**, **F** Average of the first 50% post-flash pupil diameters from 3 NHPs (MPTP2-4) for each experimental condition. The bars are presented as the mean with SD. The numbered dots represent the means of the post-flash pupil diameters for each individual flash, with numbers reflecting the flash number as presented in the pupillary response curves.

The PIPR was assessed by measuring the pupil diameter during the first 45 seconds following each of the 3 flashes per session. Comparisons were made between the averages of the experimental conditions within individual monkeys. In all monkeys, MPTP and L-dopa produced increases in the PIPR. For MPTP2, MPTP administration led to a 9.66% increase followed by a further 4.34% increase following L-dopa administration (Fig. 6A, B). In MPTP3, MPTP intoxication caused a 0.97% increase, with a further 1.25% increase following L-dopa administration (Fig. 6C, D). For MPTP4, MPTP administration led to a 4.62% increase, followed by a further 5.53% following L-dopa administration (Fig. 6E, F).

Averaging the PIPR across the three monkeys showed that MPTP administration produced a 5.00% increase when compared to preMPTP (83.80 ± 1.65% vs. 79.82 ± 2.63, *P* = 0.66, *g* = 0.87), followed by a further increase of 3.74% with L-dopa administration when compared to postMPTP (86.94 ± 3.55% vs. 83.80 ± 1.65, *P* = 0.66, *g* = 1.20). Compared to preMPTP, L-dopa administration increased PIPR significantly by 8.92% (86.94 ± 3.55% vs. 79.82 ± 2.63%, *P* = 0.04, *g* = 1.08). Hedge’s *g* scores indicate large effect sizes.

### MPTP impact on retinal thickness

**Fig. 7:**
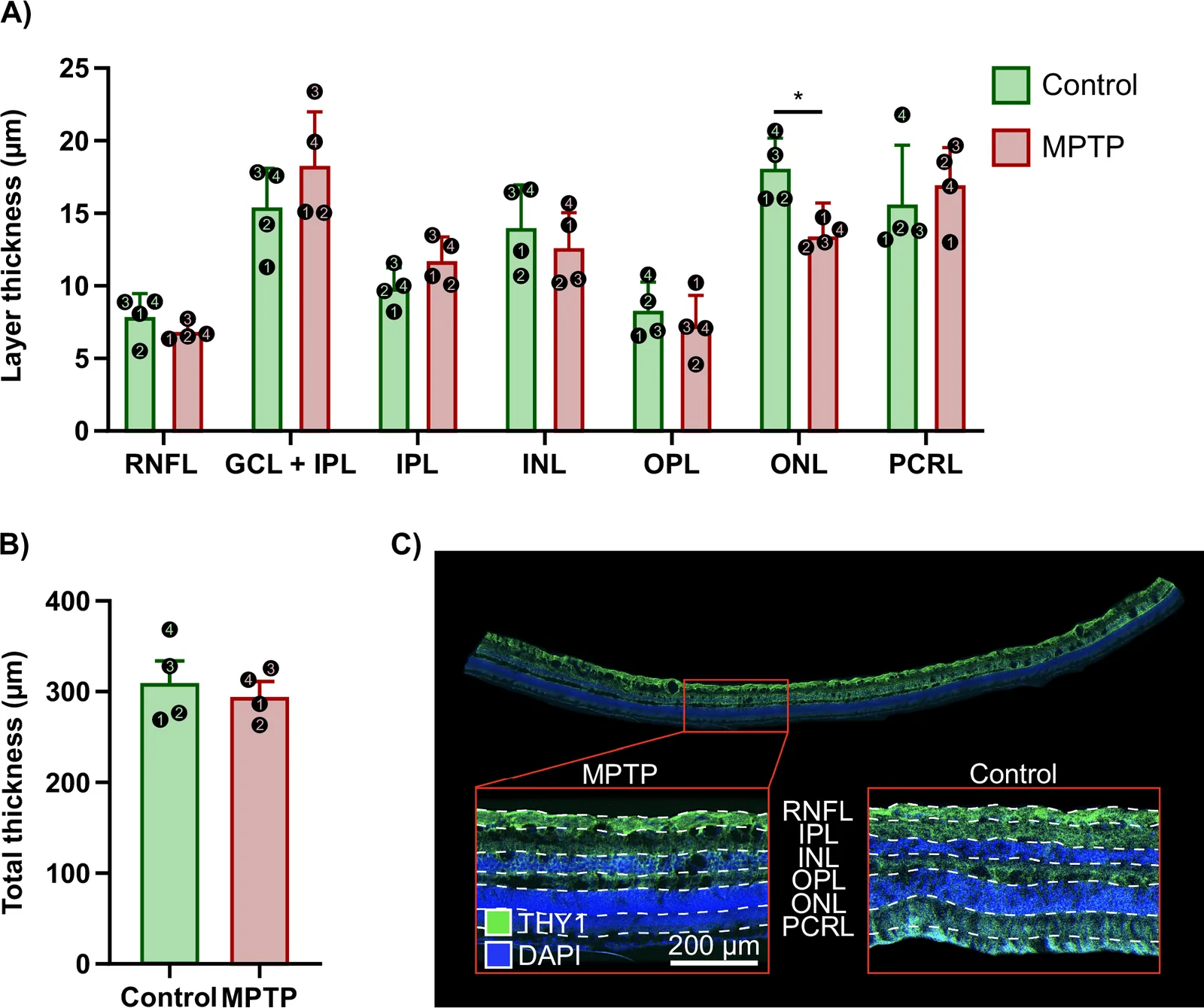
Postmortem analysis comparing retinal layer thicknesses. **A** Average thicknesses of each individual layer between control and MPTP groups. **B** Average total thickness of the retina between control and MPTP groups. The numbered dots represent the individual values with the NHP numbers as reflected in Table 1. The bars represent the mean and SD of the 4 NHPs. * = *p*-value < 0.05. **C** Fluorescent microscopy image of the cross-sectional retina illustrating the delineation of the individual layers.

To find if the functional ERG or pupillometry changes observed following MPTP administration correlated with morphological alterations of the retina, the average thicknesses of each layer of the retina were calculated for each monkey that received MPTP. Measurements were compared with control monkeys (CTL1-4) that did not receive MPTP. The data were then averaged across all monkeys of each experimental condition. The RNFL showed a -12.88% difference in thickness in MPTP-intoxicated monkeys when compared with control monkeys (13.74 ± 1.40 µm vs. 15.77 ± 3.17 µm, *g* = −0.72), the GCL + IPL showed a difference of +18.41% (36.59 ± 7.41 µm vs. 30.90 ± 5.31 µm, *g* = 0.67), the IPL showed a difference of +18.88% (23.49 ± 3.25 µm vs. 19.76 ± 2.72 µm, *g* = 1.08), the INL showed a difference of -9.88% (25.26 ± 4.83 µm vs. 28.03 ± 5.88 µm, *g* = −0.45), the OPL showed a difference of -12.13% (14.63 ± 4.05 µm vs. 16.65 ± 3.87 µm, *g* = −0.44), the ONL was significantly thinner by 25.79% (26.87 ± 4.54 µm vs. 36.21 ± 4.14 µm, *P* = 0.03, *g* = -1.87), the PRCL showed difference of +8.44% (33.93 ± 5.13 µm vs. 31.29 ± 8.08 µm, *g* = 0.34) (Fig. 7A). The thickness of the entire retina showed a -7.40% difference in MPTP-intoxicated monkeys compared with controls (143.82 ± 12.50 µm vs. 155.31 ± 23.34 µm, *g* = −0.53) (Fig. 7B). Although only the ONL thickness was significantly different, the remaining layers had Hedge’s *g* values that indicate medium to large effect sizes.

### MPTP impact on retinal dopamine cells and melanopsin expression

**Fig. 8:**
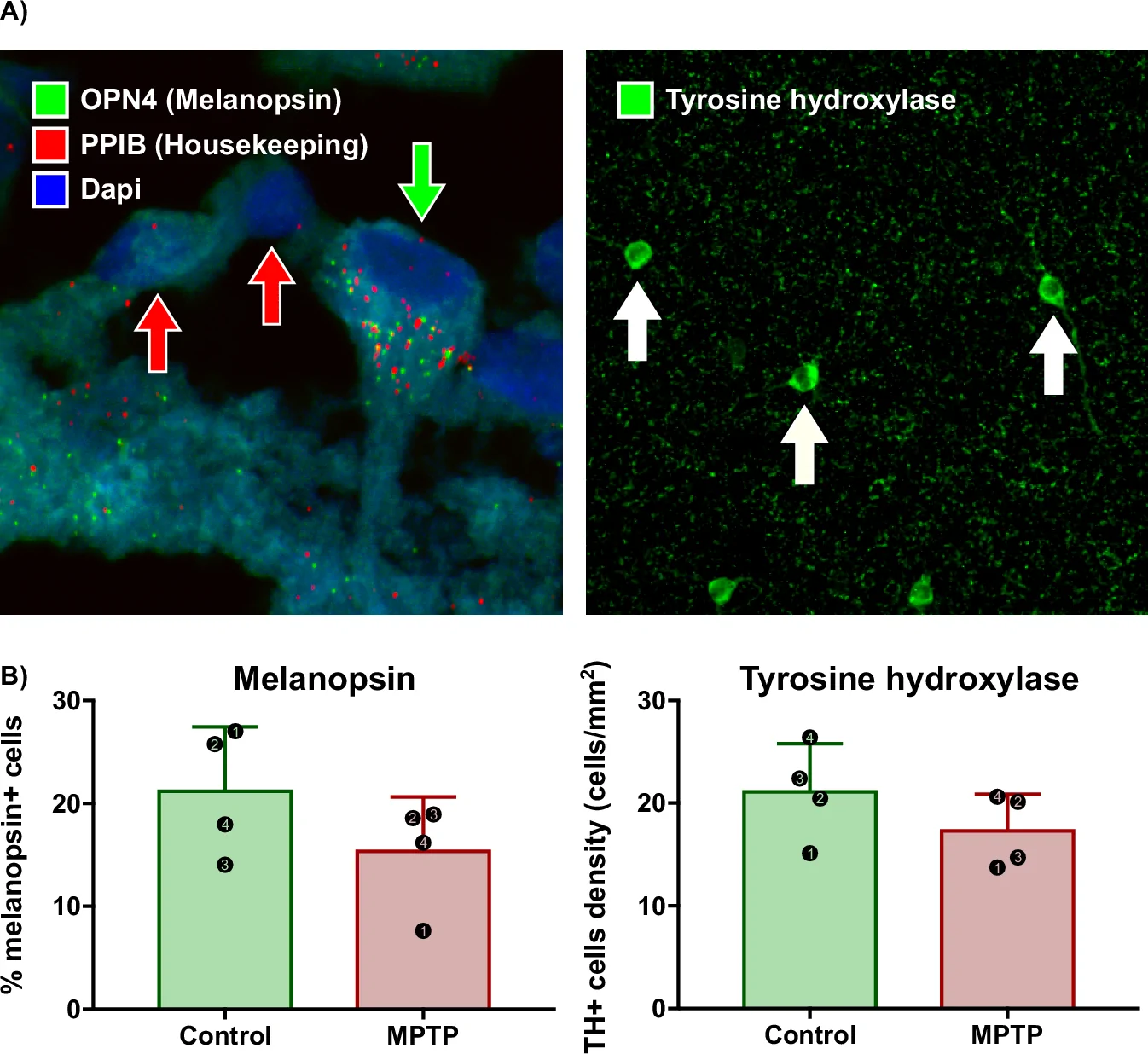
Post-mortem quantification of melanopsin and tyrosine hydroxylase (TH) expression in the retina. **A** Images captured using confocal microscopy illustrating the fluorescent staining of melanopsin RNA (OPN4) (left) and cells containing TH (right). The green arrow identifies a retinal ganglion cell containing the melanopsin RNA (OPN4), while the red arrows identify retinal ganglion cells absent of this RNA but containing the housekeeping RNA (PPIB). The white arrows identify the soma of TH+ cells. **B** Bar graphs illustrating the differences in melanopsin and TH expression between Control and MPTP animals. The bars are presented as the mean with SD. The dots represent the values of individual animals, with the numbers reflecting the NHP number as presented in Table 1.

Visual dysfunction is a non-motor symptom of PD, although the pathophysiology of PD is centred around midbrain DA neurons. Therefore, this introduces the question of whether the DA neurons of the retina are also affected in the MPTP-intoxicated monkeys. To address this question, DA cells were quantified in the retina of the four MPTP monkeys and compared to the retina of four additional control monkeys (CTL1-4). The number of TH+ cells was divided by the area to obtain cell density. The mean cell density in the retina of MPTP monkeys was 18.02% lower (17.29 ± 3.58 vs. 21.09 ± 4.69 TH+ cells/mm^2^, *P* = 0.24, *g* = −0.79) compared to controls. Melanopsin plays a critical role in pupillary reflex and its expression is thought to be affected by DA in the retina. For this reason, GCs expressing the RNA for melanopsin (OPN4) were quantified as a percentage of the overall GC count. The percentage of OPN4 RNA+ cells in the GCL of MPTP monkeys (15.34 ± 5.28%) was 27.61% lower (*P* = 0.1, *g* = −0.88) than controls (21.19 ± 6.23%, Fig. 8B). Hedges g scores indicate large effect sizes.

## Discussion

The purpose of this study was to establish PD retinal biomarkers using parkinsonian monkeys. In the first study design of its kind that we know of, ERG and pupillometry recordings were made in monkeys before and after MPTP intoxication, which is considered the gold standard animal model for PD. We found a consistent change in OP amplitudes that both reduced substantially following MPTP (Hedge’s *g* indicating small to large effect sizes) and then increased beyond preMPTP levels upon L-dopa administration (Hedge’s *g* indicating large to very large effect sizes), with OP2 reaching statistical significance under the photopic condition and trending toward significance under the scotopic condition. This highlights the sensitivity of the OP amplitudes to DA levels, and therefore, their potential as a biomarker for PD. Pupillometry recordings showed consistently increased PIPR following MPTP (Hedge’s *g* indicating a large effect size), but this was not reversed by L-Dopa administration. These in vivo results gained additional validation by post-mortem retinal evaluations, finding reduced DA AC and melanopsin-expressing RGC counts compared to control monkeys, explaining both the reduced OP amplitude and increase in PIPR. These results provide convincing evidence for two retinal biomarkers that respond to the parkinsonian condition and can be detected non-invasively using well-established techniques. These biomarkers could be valid as individual biomarkers for PD, but if taken together, they may improve the specificity of diagnosis.

OPs consist of 3 to 5 waves superimposed onto the ascending portion of the b-wave, and are thought to reflect the activity of ACs, a percentage of which are DA^34^. Therefore, OP amplitudes were evaluated to determine if MPTP affected retinal DA. From the photopic and scotopic waves, the amplitudes from 3 OPs were calculated and compared across all experimental conditions. For both photopic and scotopic recording conditions, trends toward decreases of OP amplitudes were observed, which were then reversed with the administration of L-dopa. In all cases, L-dopa produced larger OP amplitudes than the pre-MPTP condition. The same results were obtained when comparing the sum of OPs, which is used to assess the 3 OPs together. Although not statistically significant, all these changes were consistent across animals and recording sessions and the Hedge’s *g* scores indicated medium to large post-MPTP effect sizes for OP2, OP3 and the sum of OPs under the photopic condition and large to very large L-dopa effect sizes under both recording conditions (Fig. 5. Table 2).

The consistency and magnitude of the effect of MPTP and L-dopa on OP amplitudes illustrate the effect that DA has on retinal function. Indeed, modulation of DA activity has been found to directly affect OP amplitudes^64–67^. Regular exposure to tetrahydrocannabinol (THC), a known modulator of DA transmission^64^, significantly reduced amplitudes of OP2, OP3 and the sum of OP1, OP2, and OP3^66^. Administration of L-dopa, the metabolic precursor of DA, has been found to increase OP amplitudes, while drugs that reduce DA transmission have been shown to reduce them^65,67^. The activity of ACs, the primary source of DA in the retina^68^, is believed to be the main contributor to the OP waves in the ERG^34^. Indeed, in addition to having reduced OP amplitudes, the MPTP-intoxicated monkeys of the present study showed a substantial loss of TH+ ACs, highlighting a relationship between retinal DA levels and OP amplitudes. MPTP has been previously found to reduce retinal DA in monkeys and rabbits^52,69,70^, to reduce the number of ACs in monkeys and mice^55,71,72^, and to reduce OP amplitudes in mice, monkeys and rabbits^70,71,73^. Only Takatsuna et al. (1992) and Tran et al. (2022) investigated the effect of MPTP on both the OPs and DA-releasing ACs in mice, making our study the first to perform this investigation on MPTP-intoxicated monkeys. Tran et al. (2022) reported no significant reduction in OP amplitudes, despite a reduced number of DA-releasing ACs. They explained the lack of change in OP amplitudes by low MPTP dosage. Altogether, these results provide evidence that retinal and/or central DA transmission has a direct influence on OP amplitude, which decreases with lower DA content and increases with higher DA content.

It is uncertain whether the reduction in OP amplitude was caused by MPTP-induced AC degeneration, midbrain DA neuron degeneration, or a combination of both. DA releasing ACs express the DA transporter (DAT)^74^, which MPP + , the toxic metabolite of MPTP, uses to selectively enter DA cells^59^. Therefore, MPTP might directly target ACs, causing their death and diminishing their contribution to the ERG response. Some ACs also respond to DA input, possibly from central neurotransmission. Studies have found the presence of both D_1_ and D_2_ DA receptors on the membrane of ACs^68^. As such, reduced central DA could alter the activity of these cells. Indeed, Polli et al. (2021) have suggested that OPs are influenced by changes to central DA and Lavoie et al. (2014) found that modulation of central DA transmission altered the ERG, including OPs, even with no change to the local concentration of DA in the retina. It is yet uncertain how changes to central DA could affect retinal activity, but it has been hypothesized that the retinopetal pathway may be involved^75^. On the other hand, DA released from ACs might diffuse passively to reach other neighbouring DA receptor-expressing ACs, as previously proposed^76,77^, to affect their activity.

Evaluation of how OPs change in PD has recently started to gain more interest. Early studies analyzing the ERG of PD patients only showed reduced amplitude for photopic OP2 and no change for latencies^41,48^. Jaffe et al. (1987) investigated the effect of L-dopa on the ERG of PD patients, finding a significant increase in a-wave, b-wave and OP amplitudes^78^. More recent studies have investigated OP amplitudes as being a potential biomarker for early PD. Significant reductions in scotopic OP amplitudes were observed in PD patients with < 5 years of disease duration; however, these studies did not look at photopic OPs^44,46^. Patients with longer-term PD were also found to have lower OP amplitudes, despite being on L-dopa medication during the recording^43^. Collectively, these findings, along with MPTP studies, suggest that PD and MPTP can both cause reductions in OP amplitude, that are reversible when L-dopa is administered. This appears to be in agreement with the findings of the present study.

The pupil reflex following constriction is called the post-illumination pupillary response (PIPR) and is influenced by ipRGCs activity following blue light exposure. In the present study, MPTP administration led to increased average pupil diameters, indicating that the pupils were dilating at a faster rate following MPTP administration. Surprisingly, the application of L-dopa exacerbated this effect. These results were consistent for all monkeys and across recording sessions (Fig. 6). The combined sensory response from the rods, cones, and ipRGCs initiate constriction of the pupil^79^. The ipRGCs specifically slow the rate of dilation following constriction when exposed to light within the blue spectrum^49,50^. The expression of melanopsin, the active photopigment located on cellular membranes of ipRGCs, is modulated by DA^51^. Retinal DA has been found to be reduced in the retina of individuals with PD^53,54^, potentially reducing melanopsin expression and, as such, altering the PIPR. In addition, OCT studies have found thinning of the GCL and RNFL (containing the GC axons) in PD, suggesting that these cells could be less numerous, providing additional support for their potential role in the altered pupillary reflex^80^. Given that we found altered PIPR in parkinsonian monkeys along with a reduced expression of melanopsin, our results suggest that ipRGCs might be affected by DA degeneration.

These results regarding PIPR can be associated with those regarding the OP amplitude changes. ACs synapse directly onto the GCs and influence their activity^81^. In the present study, MPTP intoxication resulted in reduced OP amplitudes, suggesting lower AC activity and, therefore, reduced DA influence on the activity of ipRGCs, highlighting a potential mechanism for the altered PIPR. However, administration of L-dopa dramatically increased OP amplitudes to pre-MPTP levels in all monkeys but did not restore the pupillary reflex. One potential explanation could be that a chronic functional change of AC activity induced by MPTP could cause long-lasting changes to ipRGC activity, and that a sudden rise in DA retinal content through acute L-dopa administration was not sufficient to reverse this. As mentioned above, melanopsin expression is modulated by retinal DA, supplied exclusively by ACs^51^. As such, the reduction in OP amplitudes suggests a reduction in retinal DA content, leading to a reduced melanopsin expression. This might explain how changes in OP amplitudes could be related to changes in the pupillary reflex.

Previous studies have investigated the role of melanopsin and the ipRGCs on the PIPR in PD patients^82–85^. In these studies, a faster pupil dilation following a blue flash has been observed in patients with PD, suggesting a reduced influence of melanopsin and ipRGC on the pupil reflex. This observation was made in individuals at an early stage of PD and did not correlate with disease severity. This indicates that PIPR alteration occurs early in the disease progression, remains stable, and could therefore be used as an early biomarker for PD^82,85^. These results appear in line with those of the present study, showing that a reduced central and/or retinal DA, as observed in MPTP monkeys and patients with PD, alters the PIPR and that this alteration is likely caused by a reduced influence of melanopsin and ipRGCs.

A lower expression of melanopsin was observed in the MPTP-intoxicated monkeys (Fig. 8). This provides a potential biological substrate for the functional changes observed in PIPR following MPTP administration. Interestingly, this result closely correlates with the lower density of DA cells that we observed in the parkinsonian monkeys (Fig. 8). It has been previously established that the majority of synaptic input to the ipRGCs comes from ACs^81^, and that melanopsin expression is regulated by DA, possibly via the D_2_ receptor located on ipRGCs^51^. The results obtained from our post-mortem examination of the retina are in line with this view.

Consistent but slight increases were observed for the mean a- and b-wave amplitudes following MPTP intoxication during each of the recording conditions. However, none reached statistical significance, and Hedge’s *g* score indicated mostly small effect sizes, with the exception of the highest a-wave during the scotopic condition, indicating a medium effect size. Likewise, L-dopa did not have any effect, with results being comparable to the post-MPTP condition. These results, combined with the inconsistent findings from the previous studies, illustrate that PD has an indeterminate, and perhaps subtle, effect on the a- and b-wave amplitudes. By contrast, the b-wave implicit time was significantly delayed by MPTP during the photopic condition at Vmax, along with a trend towards a delayed a-wave implicit time, with both changes having very large effect sizes. Likewise, the scotopic pure rod a-wave implicit time trended towards a delay following MPTP. L-dopa administration produced some implicit time normalization, significantly for the scotopic pure rod b-wave and with a trend for the photopic a-wave at Vmax. The MPTP-induced delays agree with findings from PD patient studies that typically show prolonged implicit times^39,40,42,44^. While our results indicate a potential normalization of ERG implicit times following L-dopa administration, further studies with larger sample sizes are needed to confirm this effect.

Post-mortem measurements of the retinal layers indicate a statistically significant thinning of the ONL in parkinsonian monkeys, as previously reported^86^ (Fig. 7). Such a morphological change caused by MPTP may reflect those previously observed in patients with PD. Using optical coherence tomography (OCT), an imaging technique that noninvasively constructs cross-sectional images of tissue, previous studies have provided data on the thickness of retinal layers in PD patients that were recently compiled into a meta-analysis^80^. This report indicates a significant thinning of the RNFL and the GCL + IPL in PD subjects. Other layers showed no significant difference in thickness. Interestingly, only two studies reported the thickness of the ONL, which both showed a reduction in thickness^87,88^, in line with the present study. More attention should certainly be paid to the ONL when measuring retinal layer thicknesses in PD patients using OCT, as it appears to be a layer affected by PD that has so far been underreported.

The ONL contains the soma of photoreceptor cells, so an MPTP-induced thinning of this layer implies that they might be reduced in number. The expected outcome of such a change would be a reduction in the a-wave amplitude. Our results rather showed consistent increases of the a-wave amplitude. This could be explained by compensatory mechanisms of the remaining rods and cones. Currently, very few studies have investigated the ERG in PD and made comparisons to retinal layer thicknesses. These studies found either no difference in retinal structure or no correlation to disease duration or severity, despite finding differences in ERG^40,44^. It was concluded that changes to ERG likely occur before structural changes and, therefore, appear as more reliable biomarkers for PD diagnosis. More studies are needed to establish correlations between physiological and morphological disease-induced changes to the retina.

One challenge with using NHPs as experimental subjects is their availability. Not only are they difficult to acquire and expensive to house and care for, but ethical limitations also ensure that as few animals are used as possible. This meant that there was limited availability of subjects for both the in vivo tests, reducing the statistical power, and for control subjects needed for the post-mortem comparisons. For this reason, the control subjects that we could acquire did not match for sex and age. Therefore, it is difficult to discern if the post-mortem differences were caused by MPTP or if these confounding variables may have influenced our data. A larger sample size would be needed to assess any sex or age-related differences. Sex differences have been found in the retinal thickness in cynomolgus monkeys, potentially explaining the difference observed in the present study^89^. Regarding the difference in age of the animals, retinal development is largely complete by birth, with only rearrangement and elongation of photoreceptors occurring postnatally and primarily in the fovea, whereas cells in the perifovea, from which measurements were taken, are largely unchanged^90,91^. Furthermore, macaque retinal cell redistribution has been found to reach full maturity by 15 months^92,93^. By comparison, thinning of the macaque RNFL, thought to represent overall retinal thinning, has been found to occur at a rate of 0.70 μm per year from birth, illustrating that age-related thinning between the groups should be negligible, particularly for the difference seen in the ONL^94^. Therefore, it is unlikely that age would have had a major effect on the differences observed in retinal thickness and the density of ACs.

The results of the present study would be strengthened by future work. Firstly, statistical significance would be essential for validation of the results as PD biomarkers, and this was presently limited by the small sample size and the consequent low statistical power. This was especially apparent for the PIPR, which was further limited by only having 3 available subjects, and with just 2 out of 3 subjects showing a convincing post-MPTP difference. Despite this limitation, the large effect sizes observed suggest that additional subjects would likely yield statistically significant biological differences. Furthermore, while MPTP is considered the gold standard as an animal model for PD, it is difficult to confirm whether the observed changes resulted from the lesioning of SNc DA neurons or from alternative adverse effects of the neurotoxin, given its systemic administration. As such, the multimodal approach presented in this study should be performed on other PD animal models that directly target SNc DA neurons and allow for within-subject comparison, such as intracerebral 6-hydroxydopamine injections, or in human PD patients, to confirm if the retinal changes are translatable. Finally, while the goal of the present study was to find convincing retinal biomarkers to aid PD diagnosis, it is hoped that these biomarkers could be used for early, prodromal diagnosis. To confirm this, recordings should be done in an animal model that mimics the pre-motor symptom stage of the disease. This could be achieved by using a lower dose of MPTP, or by using the same protocol but with recordings being made immediately following initial administration and throughout the degeneration process.

To our knowledge, this is the first study to take such a multimodal approach towards finding retinal biomarkers of PD. Comprehensive comparisons were made between many metrics within and across several different techniques. From ERG, we compared a-wave, b-wave, PhNR, and OP amplitudes and latencies, along with both scotopic and photopic conditions to elucidate how the rod and cone systems are differentially affected. Evaluations were made both ON and OFF L-dopa medication to better understand its potential impact on ERG normalization. Previous studies tended to look only at specific waves or light conditions and did not compare the effect of medication. In the future, it would be beneficial for similar studies to comprehensively incorporate all aspects of the ERG, under both photopic and scotopic conditions, comparing with and without medication so that more apparent and cohesive results can be obtained. In doing so, there will be a greater possibility to come to a clear consensus on how PD affects the ERG and, therefore, retinal activity. Furthermore, this is the first study to investigate pupillometry alongside ERG. The biggest challenge in finding a retinal biomarker for PD is to confirm its specificity for the disease. Although differences in ERG signatures have been reported in PD, these changes may be too subtle or insufficiently specific to support diagnosis on their own. Evaluating specificity is difficult as current research investigating ERG and PIPR changes in other neurological conditions is lacking^20^. Increased a- and b-wave implicit times are reported in other conditions, including autism spectrum disorder, schizophrenia, bipolar disorder, major depressive disorder, and Alzheimer’s disease^20^. Reduced OP amplitudes have been found in schizophrenia^95^, as well as impaired PIPR^96^, which has also been implicated in other α-synucleinopathies and REM-sleep behaviour disorder^97–99^. However, OP amplitudes and PIPR appear to be unaffected by Alzheimer’s disease^100,101^. Future research developments may find more similarities with other conditions. Consequently, identifying a more robust signature through the integration of multiple techniques and complementary biomarkers is essential. This multimodal approach is particularly critical for the development of early biomarkers, when the clinical features traditionally used for diagnosis are not yet fully expressed. By performing pupillometry in addition to ERG, we were able to establish a potential link between PIPR and OP amplitude, further supported by our post-mortem data. Hopefully, this will inspire future studies to use a similar approach towards finding other combinations of markers from multiple recording techniques that together can form specific signatures for given diseases.

To conclude, this study highlights the changes that occur to the ERG and pupillary reflex following the DA loss that characterizes PD. Measurements were compared within animals, reducing the impact of interindividual variability. Among the observed outcomes, OP amplitudes and the PIPR appear to present the most convincing changes in response to DA loss, indicating their potential as sensitive retinal biomarkers for PD. This is further strengthened by their connection to retinal DA, which is known to be reduced in PD and in MPTP-intoxicated monkeys. Therefore, combining these retinal biomarkers with additional measures previously reported, such as alterations to retinal spectral recordings using diffuse reflectance spectroscopy^86^, may contribute to the development of a more specific diagnostic signature for PD. Such a multimodal approach could ultimately improve diagnostic accuracy and facilitate earlier detection, including during prodromal stages.

## Methods

### Animals

This study was conducted using four female cynomolgus monkeys (*Macaca fascicularis*), all aged 5 years old and weighing between 3.2 and 3.6 kg (MPTP1-4, Table 1). Ovariectomy was performed on these animals to model the hormonal status of women with menopause. This is particularly relevant to the fact that most parkinsonian women are menopausal and that oestrogens are known to be neuroprotective^57^. Animals were housed under a 12-hour light-dark cycle with unlimited access to food and water. Experiments were approved by the Comité de Protection des Animaux de l’Université Laval (approval number: 2019-314), in accordance with the Canadian Council on Animal Care’s guide to the Care and Use of Experimental Animals. The minimum necessary number of animals was used to complete this study.

Eight additional monkeys (*Macaca fascicularis*) were used as controls for post-mortem comparisons. Four were used for post-mortem analysis of the retina (CTL1-4, Table 1) and four others were used for post-mortem analysis of the brain (CTL5-8, Table 1). Unless stated otherwise, numbered dots presented in figures correspond to monkey identifications as listed in Table 1.

### Procedure and timeline for ERG and pupillometry recordings

Prior to the procedure, animals were anesthetised using ketamine (10 mg/kg) with dexmedetomidine (0.4 mg/kg). Additional dosages were made at regular intervals during the procedure to prolong the anaesthesia as necessary. Isoflurane was not used as it can interfere with ocular recordings^58^. Efforts were made to minimize the duration that the animals were under anaesthesia. Pupillometry was performed on the left eye. During this recording, the left eye was held open using an eye speculum and the right eye remained close to prevent drying. Throughout all recordings, moisturizing eye drops were applied to the open eye regularly. Pupillometry recordings were made using the RETeval device (LKC Technologies). Recordings were made in complete darkness to minimize the influence of ambient light on the pupil diameter. The device was placed over the eye and the iris was positioned inside the digital circle overlay on the device screen, within which the boundary of the pupil is automatically detected. The pupil diameter was continuously recorded during a sequence of three 1-second blue-light flashes, each followed by 90 seconds of darkness. A baseline pupil diameter was established by recording the pupil 5 seconds prior to the first flash.

Upon completion of the pupillometry and prior to ERG recordings, both pupils were dilated with tropicamide (1%) and phenylephrine (2.5%). In preparation for the ERG, the monkey was positioned on its back with its eyes directed towards the ceiling. The eyelids were held open with surgical tape. The recording Dawson-Trick-Litzkow (DTL) electrodes (Shieldex 33/9 Thread, Statex, Bremen, Germany) were carefully placed on the surface of each eye. Subcutaneous reference electrodes and a ground electrode were placed respectively on the lateral side of each eye (outer canthus) and between the eyebrows. The Ganzfeld Colordome (Diagnosys LLC, Lowell, MA, USA) was positioned above the face of the monkey, so that the eyes were in the centre of the opening and were completely encompassed. ERG recordings were acquired using Espion E3 software (v5.0.45; Diagnosys LLC, Lowell, MA, USA). In accordance with ISCEV standards, band-pass filters were set to 0.15–300 Hz for standard ERG recordings and to 75–300 Hz for OP recordings. Photopic ERGs were recorded under steady white background illumination (30 cd/cm^2^) provided by the Ganzfeld. Light adaptation was made for 5 minutes in order to saturate the rods and therefore measure the cone response only^25^. Photopic ERG recordings were triggered by 14 light intensities ranging from −2.2 to 2.9 Log cd·s·m^−^^2^. White LED flashes (6500 K) were used for stimulus intensities up to 1.40 log cd·s·m⁻², while higher intensities were delivered using a white xenon source. For each flash intensity, the average response of 15–20 flashes was calculated. The inter-flash interval was set to 5 seconds for flash intensities up to 0 log cd·s·m⁻² and to 15 seconds for higher intensities. Following completion of the photopic protocol, the PhNR protocol was performed using a steady blue background illumination (LED, B472, 30 cd/m²) and four red LED flash intensities ranging from 0.18 to 2.82 cd·s·m⁻² and presented at a 1-second interval, in accordance with previously established optimal conditions^32^. Given the relatively small amplitude of the PhNR, responses were averaged over 80 flashes at each flash intensity to improve the signal-to-noise ratio. Finally, scotopic ERG recordings were performed following 20–30 minutes of dark adaptation. During the adaptation period, a dim flash stimulus (−2.0 log cd·s·m⁻²) was presented every 3 minutes and ERG responses were monitored. Dark adaptation was considered complete when the ERG b-wave amplitude remained consistent for two consecutive assessments, typically taking place between 20 and 30 minutes of adaptation. Scotopic recordings were obtained using light flashes presented at 15 intensities ranging from −3.4 to 2.9 Log cd·s·m^−^^2^. Flashes were achieved using white LED (6500 K) up until 1.40 Log cd·s·m^−2^ from which white xenon was used. For each intensity, the average response of 5-10 flashes was calculated. Flash interval was 5 seconds up to the 0 Log cd·s·m^−2^ and 15 seconds for the remaining flash intensities.

Two blocks of 3 recording sessions were performed, with MPTP administration immediately after the first block. The second block began 5 months later to allow the complete induction of stable Parkinsonian motor symptoms. During each block, recording sessions were separated by 2-week intervals. Each recording session occurred over 2 full days, with half of each day being devoted to 1 of the 4 monkeys (Fig. S1). At the end of each recording session, cerebrospinal fluid (CSF) samples were taken from which the concentration of the DA metabolite homovanillic acid (HVA) was assessed using high-performance liquid chromatography with electrochemical detection^59^.

### MPTP administration

Immediately following the third session, MPTP (product no. M0896, Sigma) was delivered at a concentration of 8.61 mg/ml via a subcutaneous osmotic mini-pump continuously for 14 days. Additional intramuscular MPTP was injected as required until stable parkinsonian symptoms had been established. The mean total MPTP delivered was 11.54 ± 2.23 mg (Table 1). The scoring of parkinsonian symptoms was made using a scale developed specifically for NHPs that assesses the behavioural response to MPTP for posture, mobility, climbing, gait, grooming, vocalization, social interaction and tremor^60^. Assessments were made every 15 minutes during a 2-hour period on 2 consecutive days, the week prior to the post-MPTP block of recording sessions.

### Levodopa administration

Approximately 30 minutes prior to the seventh recording session, the monkeys were treated with levodopa (L-dopa) to evaluate the effect of an acute elevation of systemic DA on ERG and pupillometry responses. This was administered by intramuscular injection at a concentration of 25 mg/kg with 50 mg of carbidopa, a peripheral inhibitor of the aromatic L-amino acid decarboxylase enzyme. The seventh recording session was performed as previously described.

### Euthanasia

The monkeys were euthanized 1 day following the final recording session. Transcardial perfusion was performed with 500 mL of phosphate-buffered saline (PBS, 0.1 M, pH 7.4) followed by 1.5 L of paraformaldehyde 4% (PFA) with 0.2% of glutaraldehyde, and finally 1 L of PFA 4%. Each of these solutions was kept on ice during delivery. Once complete, the brain was removed from the skull and placed in PFA 4% for 24 hours before being placed in PBS and stored at 4 °C. These brains were then sliced in 50 µm-thick sections using a vibratome, with sections being stored in an antifreeze solution (PBS, ethylene glycol, glycerol - 4:3:3 ratio) with sodium azide (0.1%) at -40°C. Both eyes were also removed, and an incision was made on the cornea before being placed in 4% PFA.

### Retinal dissection and preparation

Post-mortem analyses were performed on the retina of the 4 MPTP-intoxicated monkeys, in addition to the retina of 4 additional controls (Table 1). Eyes were all kept in 4% PFA for 7 days before being transferred to either PBS (left eyes) or a solution of sucrose (30%) with sodium azide (0.1%, right eyes) for cryoprotection. To dissect the retina, the cornea and optic lens were removed first. The eye was oriented using the optic nerve as a landmark before four equally spaced cuts were made from the opening of the cornea towards the macula. The eye was spread flat with four “wings” at dorsal, ventral, medial and lateral positions and the macula and optic nerve head (ONH) at the centre. Using a brush, the retina with the epithelial layer was gently detached from the sclera. The 4 wings were trimmed to create flat edges before each was divided into 3 equal quadrilateral sections. A cut was made on the left corner of the most distally located edge from the macula for orienting the section. They were then placed into individual wells containing PBS (left eye) or the sucrose solution (right eye) and stored at 4 °C until use. The most proximal sections to the macula within the medial wing were selected from both eyes for analysis. The epithelial layer was removed from the retina as it was found to interfere with visualization. Retinal sections from the right eye were sliced perpendicular to the macula into 14 µm-thick transverse sections with a cryostat at -20°C and collected on microscope slides. Retinal sections from the left eye remained intact and were used as a flat mount.

### TH immunohistochemistry and Cresyl Violet staining of the SNc

To quantify the extent of the MPTP-induced DA neuronal lesion in the SNc, immunohistochemistry was performed in 5 equally-spaced transverse sections (850 µm interval) taken within the range of -5 and -10 mm from the anterior commissure according to a brain atlas^61^. Incubations were made at room temperature with agitation unless stated otherwise. Free-floating midbrain sections first underwent peroxidase neutralization by incubating in a solution consisting of 45% ddH_2_O, 45% ethanol and 10% H_2_O_2_ for 30 minutes followed by 5 × 5-minute washes in PBS. They were then incubated in a blocking solution consisting of 2% normal donkey serum (NDS) and 1% TritonX-100 diluted in PBS for 1 hour. This same blocking solution was used to dilute the primary antibody against TH made in rabbit (1/1000, product no. AB152, EMD) within which the sections were incubated overnight. Following a PBS wash, sections were incubated for 1 hour in biotinylated horse antibody against rabbit (1/400, product no. 711-065-152, Jackson) diluted in blocking solution. Following another PBS wash, sections were incubated in avidin-biotin-peroxidase complex diluted in PBS (1/100, product no. PK-6100, Vector Laboratories) for 1 hour. Prior to 3,3’diaminobenzidine tetrahydrochloride (DAB, product no. D5637, Sigma-Aldrich) staining, sections were washed first in PBS and then in TRIS. DAB was diluted in TRIS (0.025%) along with 0.005% ddH_2_O_2_ and sections were incubated in this solution for approximately 4 minutes until sufficient staining could be observed. The reaction was stopped by washing first in TRIS and then PBS. The sections were then mounted on gelatin-coated slides and left to dry overnight before dehydration in 70% ethanol for 10 minutes, rehydration in ddH_2_O for 5 minutes, and staining in Cresyl Violet for 25 minutes followed by a brief rinse in ddH_2_O. Finally, sections underwent graded dehydration in ethanol (70%, 95%, 100% x 2), were cleared with toluene and then coverslipped with permount.

### TH immunohistochemistry of the striatum

To quantify the extent of MPTP-induced DA denervation in the striatum, immunohistochemistry was performed in 3 transverse sections taken -3 mm, 0 mm, and 3 mm from the anterior commissure^61^. Incubations were made at room temperature with agitation unless stated otherwise. Free-floating sections were first incubated in a blocking solution consisting of 2% normal goat serum (NGS) and 0.1% TritonX-100 diluted in PBS for 1 hour. This same blocking solution was used to dilute the primary antibody against TH made in rabbit, within which the sections were incubated overnight at 4 °C. Following a PBS wash, sections were incubated for 1 hour in biotinylated goat antibody against rabbit (1/200, product no. BA 1000, Vector Laboratories) diluted in blocking solution. Following another PBS wash, sections were incubated in infrared probe (800 nm) coupled streptavidin diluted in PBS (1/1000, product no. 926-32,230, LI-COR Biosciences) for 1 hour. After a final PBS wash, the sections were mounted on gelatin-coated slides and left to dry before coverslipping with fluorescent antifade mounting medium (DAKO, product no. S3023, Carpintria, CA, USA).

### Retinal immunohistochemistry

To measure MPTP-induced structural differences to the retinal layers, immunostaining was performed for the proteins Thy1 (1/800, product no. AB181469, Abcam) and DAPI (product no. D-1388, Sigma). Microscope slides containing cross-sections of the right retina were initially washed in a bath of PBS 3 times for 5 minutes each before being incubated for an hour in a blocking solution of PBS with 2% normal donkey serum (NDS) and 0.1% Triton X. Thy1 antibody was diluted in the same blocking solution, in which the slides were incubated overnight at 4°C. The next day, the sections were washed in PBS before incubating for 2 hours in the secondary antibody 488 anti-mouse (1/400, product no. 715545150, Jackson) diluted in blocking solution. After another PBS wash, the sections were covered in DAPI solution for 10 minutes before a final PBS wash. The slides were then coverslipped using Dako fluorescent mounting medium (product no. 53023, Agilent).

To quantify ACs, immunostaining was performed for TH and DAPI. Flatmount sections of the left retina were placed in wells and were washed in PBS 3 times for 5 minutes each. Sections were then incubated in a blocking solution of PBS with 2% NDS and 2% Triton X followed by incubation in TH antibody diluted in the blocking solution for 72 hours at 4 °C. Sections were washed again in PBS before incubating for 24 hours in the secondary antibody 488 anti-rabbit (1/400, product no. 711545152, Jackson) diluted in blocking solution. Following a PBS wash, DAPI was then applied to the sections for 10 minutes before a final PBS wash. The slides were then coverslipped using Dako fluorescent mounting medium.

### Retinal RNAscope

For quantification of melanopsin-containing GCs, RNAscope was performed to stain for the OPN4 RNA (translates to the melanopsin protein) (Product no. 442211, Advanced Cell Diagnostics), and PPIB (housekeeping RNA present in all cells) (Product no. 313911-C3, Advanced Cell Diagnostics) using the RNAscope kit from Advanced Cell Diagnostics (Product no. 323100) on the cross-sections of the right retina. The RNA probes and washing buffer were first placed at 40°C for 10 minutes, and 10X Target Retrieval Reagents were diluted 1:10 in ddH_2_O to produce 1X Target Retrieval solution, which was brought to a boil. The retina slides were placed in the boiling Target Retrieval solution for 5 minutes after washing in PBS for 5 minutes. The sections were then briefly rinsed in ddH_2_O twice and 100% ethanol once before leaving to dry. RNAscope Protease III and IV were dropped onto the sections until covered and left to incubate for 20 minutes at 40°C. Afterwards, the sections were put in a container with sufficient moisture so that they don’t dry out for the following steps. The C1 (OPN4) and C3 (PPIB) probes were mixed together at a ratio of 50:1 and dropped onto the slides so that all sections were covered. They were then incubated for 2 hours at 40°C before being washed twice with wash buffer for 2 minutes with agitation. Signal amplification was made by performing 4 incubations at 40°C using the 4 amplification solutions, with 2 washes in wash buffer for 2 minutes with agitation following each incubation. Incubation times were 30 minutes for the Amp 1 FL solution, 15 minutes for Amp 2 FL, 30 minutes for Amp 3 FL, and 15 minutes for Amp 4 FL. The fourth amplification solution determines the fluorophore labelling. In this case, Amp 4 Alt A-FL was used to label OPN4 with Alexa 488 and PPIB with Atto 647. Following amplification, sections were covered in DAPI for 10 minutes, rinsed in ddH_2_O, and then coverslipped using Dako fluorescent mounting medium.

### Quantification of TH+ neurons in the SNc

To quantify the TH+ cells in the SNc, live stereological analysis of the midbrain sections stained for TH was performed using a light microscope connected to a computer running Stereoinvestigator software (v. 7.00.3, MicroBrightField, Colchester, VT, USA). Quantification was performed using the Optical Fractionator probe within the software. On each of the 5 transverse sections per monkey, the SNc was contoured with the help of a brain atlas^61^. A grid of 800 × 800 µm dimensions was randomly placed over the defined contour. At the upper left intersections of this grid, 250 × 250 µm counting frames were placed. Using a 40x objective, TH+ cell bodies that came into focus within a 20 µm-thick optical dissector were counted if their nuclei fell within the counting frame and/or were touching the inclusion lines. Averages of 286 ± 45 and 42 ± 23 TH+ neurons were counted in the SNc of control and MPTP-intoxicated monkeys, respectively. Coefficients of error (Gunderson m = 1 and 2nd Schmitz-Hof) ranged between 0.06 and 0.29. The mean measured SNc section thicknesses were 23.29 ± 1.33 µm and 23.43 ± 1.21 µm for control and MPTP-intoxicated monkeys, respectively.

### Quantification of TH immunoreactivity in the striatum

The LI-COR infrared imaging system was used to scan the entirety of all immunostained sections, producing an image illustrating the extent of staining throughout the tissue. The striatum of each section was divided into its functional territories according to the work of Parent and Hazrati (1995). The 3 sections were divided into the sensorimotor and associative functional territories for the caudate nucleus and putamen. For each of these regions of interest, the software generated an optical density value, representing the intensity of infrared staining for TH.

### Quantification of retinal layer thickness

To measure the thicknesses of the retinal layers, images were acquired from each monkey’s retina using a slide scanner fluorescent imaging system (TISSUEscopeTM 4000, Huron Technologies, Waterloo, Ontario, Canada) and loaded into the ImageJ software. The metadata of the images provided their dimensions in micrometre, which we could use to calibrate the scale in ImageJ. Using the straight-line drawing tool, contours were drawn around each region of interest representing each layer of the retina. The area and perimeter measurements were obtained and used to calculate the width and length of an equivalent rectangle. The width of this rectangle was considered the thickness of the retinal layer. The regions of interest measured within the retina were the retinal nerve fibre layer (RNFL), the ganglion cell layer (GCL) and inner plexiform layer (GCL + IPL), the inner plexiform layer (IPL), the inner nuclear layer (INL), the outer plexiform layer (OPL), the outer nuclear layer (ONL) and the photoreceptor cell layer (PRCL). The entire thickness of each retina was also measured.

### Quantification of TH+ cells in the retina

To quantify the TH+ ACs in the retina, tile-scan images were first obtained from each flatmount section using a confocal microscope combined with the Stereoinvestigator software. Using the 20x objective, the laser intensity and brightness were adjusted to maximize the appearance of the cells while minimizing background noise. Image resolution was set to 512 × 512 pixels, and pinhole size was set to 3.2 µm (1 airy unit). Stereoinvestigator was opened, and a custom-made macro was used to connect with Zen. By setting the lowest magnification and using the brightfield camera, the section was visualized on Stereoinvestigator, in which the contour was outlined. Then the SRS Image Stack Workflow was initialized. Magnification was set to 20x, the thickness was set to 50 µm, as this had been previously determined to contain all of the tissue, and the step size was set to 3 µm. Adjacent z-stacks were acquired, for which the top of the section was automatically determined by Stereoinvestigator controlling the confocal stage. Using Stereoinvestigator and the obtained tile scans, TH+ ACs were quantified through the entire thickness of the tissue. The number of cells counted was divided by the area of the region of interest to provide the density of TH+ ACs.

### Quantification of melanopsin RNA in the retina

To quantify the number of GCs containing melanopsin, tile scans were obtained of the cross-sections using the same technique as mentioned above. In this case, the objective used was 63x. The tile scans were opened in Stereoinvestigator, and all retinal GCs were clearly identified using the DAPI staining and counted using a specific identification marker. A second identification marker was used for GCs that contained the stained melanopsin mRNA puncta. To minimize false positives, cells were only counted if they contained at least 2 puncta for melanopsin mRNA or 1 large aggregate.

### Analysis of ERG and pupillometry recording data

The ERG waveform consists of an initial negative component (a-wave) followed by a larger positive component (b-wave). A third component, the photopic negative response (PhNR), occurs after the b-wave and is optimally elicited using a red flash on a blue background. By convention, the a-wave amplitude is typically measured from the baseline, and the b-wave amplitude is measured as the difference between the trough of the a-wave and the peak of the b-wave. The PhNR amplitude can be taken from either the baseline or from the peak of the b-wave. Implicit times to each peak and trough amplitude are measured from the flash onset. Along the ascending limb of the b-wave are the OPs, which appear as 3 to 5 consecutive wavelets. They can be isolated from the rest of the ERG by adjusting the recording bandpass from 0.15–300 Hz to 75–300 Hz. The peak amplitude can be taken from each individual OP wave or added together to give the sum of OPs.

The recorded ERG waves were cleaned by first removing the few that contained artifacts due to eye or eyelid movement. The remaining ERG waves were averaged for each flash intensity. Peak and trough amplitudes, as well as implicit times, were automatically estimated by the Espion software and manually adjusted when necessary. For the PhNR, the recording protocol was optimized during the first two preMPTP sessions, and the finalized protocol was applied from the third session onward. Consequently, only PhNR data from the third preMPTP session onward were included in the analysis.

All ERG data were analyzed separately for photopic and scotopic conditions, with comparisons made between preMPTP, postMPTP, and L-dopa conditions. Amplitudes and implicit times of the a- and b-waves were averaged between the left and right eyes of each monkey, for each flash intensity, and for all recording sessions of each condition. Average a-wave and b-wave amplitudes were plotted to generate their respective photopic and scotopic luminance-response functions. From these functions, several parameters were derived, including Vmax (maximum combined a- and b-wave amplitudes) and the scotopic pure-rod amplitude. For the photopic luminance response function, Vmax was defined as the maximum b-wave amplitude observed across all flash intensities. However, because the flash intensity at which Vmax occurred varied between animals, we selected the two specific intensities at which Vmax was reached in the monkeys (0.4 and 0.6 Log cd·s·m^-2^) and averaged the corresponding responses. Implicit times from these same intensities were also averaged, yielding a single Vmax amplitude and implicit-time value for each animal. Similarly, the highest a-wave amplitude was defined as the largest a-wave amplitude observed across all flash intensities. Similarly, for the scotopic ERG, the b-wave Vmax was obtained by averaging the four flash intensities at which the maximum b-wave amplitude was observed (0, 0.4, 0.6, and 1 Log cd·s·m^−2^). The pure rod response a-wave and b-wave amplitude and implicit time were taken at a fixed intensity of -1 Log cd·s·m^−2^. The same process was followed to produce the PhNR luminance response curve, from which the maximum amplitude was selected for further analysis.

The OP data selected for analysis were from the flash intensities that produced the waveforms with the largest apparent amplitudes. These corresponded to 0.40 Log cd·s·m^−2^ for photopic and 1.40 Log cd·s·m^−2^ for scotopic conditions. The raw OP wave data for each individual animal were averaged for the left and right eyes and for all recording sessions of each experimental condition. From the averaged data, the peaks of each OP were obtained. For consistency, across all conditions, the peaks of only the first 3 OPs were used. The amplitudes of these individual OPs were averaged across the 4 animals for each experimental condition. In addition, for each animal, the sum of the amplitudes of the 3 OPs was calculated for each condition. The mean of these sums of OPs was then calculated across the 4 animals. The mean OP waveforms of the 4 animals were used for visualization.

Analysis of the pupillometry data was made by comparing pupil diameter as a function of time in response to blue light flashes and comparing values between preMPTP, postMPTP and L-dopa conditions. Pupillometry data extracted from the RETeval device included the raw data of pupil diameter measured in millimetre (mm) by time of recording measured in seconds (s). The device recorded at 30 Hz. Three 1-second blue light flashes were delivered at time 0 s, 90 s and 180 s. Firstly, the time points of the lowest recorded diameters following each post-flash period were attained. The baseline pupil diameter was calculated by averaging all diameters for the 5 seconds prior to the first flash, from which all other diameters were normalized as a percentage. A 5-second moving average of the pupil diameters was calculated to reduce noise. To visualize the data, the waves were averaged by experimental condition for each monkey. The pupil of MPTP1 only partially dilated following each flash, therefore making it impossible to analyze, and was excluded from further analysis. The remaining 3 monkeys showed normal pupil reflexes and were included for analysis. To quantify post-illumination pupillary response (PIPR), all diameter values between the lowest diameter post-flash and the 45-second post-flash timepoint (first 50% of post-flash values) were averaged for each flash of each experimental condition. Each of these values was then averaged by experimental condition for each animal and then together to provide the final averages.

### Statistical analysis

Statistical analyses were performed using Graphpad Prism (v. 9, Dotmatics, San Diego, CA, USA). All within-subject comparisons for the in vivo data were performed using the Friedman test with Dunn’s post-hoc test for multiple comparisons. All comparisons between the control and MPTP groups for the post-mortem data were performed using unpaired, non-parametric t-tests (Mann-Whitney). Significance was considered if *P* < 0.05. To aid interpretation of the results, given the small sample size, we calculated the Hedge’s *g* effect sizes (bias-corrected standardized effects). Within-subject comparisons were calculated as the standardized mean change using the standard deviation of the paired difference scores. Between-group comparisons were calculated as the standardized mean difference using the pooled standard deviation. Effect size scores 0.2 – 0.5 were considered small, 0.5 – 0.8 were considered medium, 0.8 – 1.3 were considered large and > 1.3 were considered very large^62,63^. Unless otherwise stated, values are represented as the mean with standard deviation.

## Supplementary information

## Data Availability

The raw datasets generated and/or analysed in this study are publicly available on Figshare: https://doi.org/10.6084/m9.figshare.31817110.
