# Supplementary material for: Improving Parkinson’s disease diagnosis by non-invasive detection of retinal biomarkers in MPTP monkeys using ERG and pupillometry
Source: NPJ Parkinsons Dis. 2026 Jun 3;12:211. doi: 10.1038/s41531-026-01391-y (PMC13538655; doi:10.1038/s41531-026-01391-y)
Supplement: Supplementary file 1 [file 41531_2026_1391_MOESM1_ESM.pdf]

Supplementary material

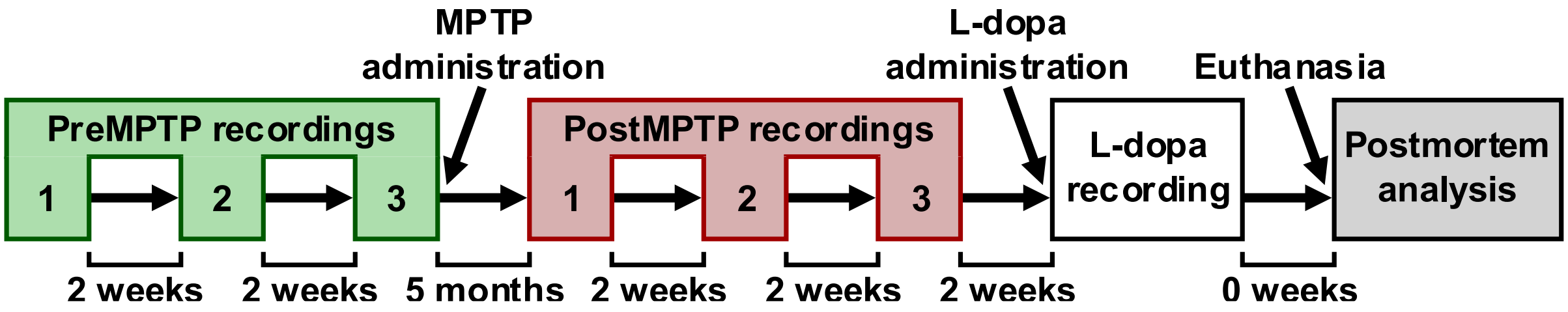

Figure S1: Experimental timeline. Each recording session occurred over 2 days with half a day devoted for each NHP. Postmortem analyses were performed on a single retina from each animal and compared to single a retina from 4 control NHPs.
